# A GMP-compliant formulation of regeneratively active polyphosphate for wound healing and skin regeneration

**DOI:** 10.1039/d6bm00151c

**Published:** 2026-04-24

**Authors:** Werner E. G. Müller, Meik Neufurth, Xiaoqin La, Hadrian Nassabi, Mathias Brösicke, Rita Dobmeyer, Rafael Muñoz-Espí, Changxin Wu, Hiroshi Ushijima, Heinz C. Schröder, Xiaohong Wang

**Affiliations:** a ERC Advanced Investigator Grant Research Group at the Institute for Physiological Chemistry, University Medical Center of the Johannes Gutenberg University Duesbergweg 6 D-55128 Mainz Germany wmueller@uni-mainz.de wang013@uni-mainz.de; b Institutes of Biomedical Sciences, Key Laboratory of Chemical Biology and Molecular Engineering of Ministry of Education of China and Key Laboratory of Medical Molecular Cell Biology of Shanxi Province, Shanxi University No. 92 Wucheng Road 030006 Taiyuan China; c Department of Dermatology with Plastic Surgery, SRH Wald-Klinikum Gera GmbH Friedens Street 122 D-07548 Gera Germany; d Department of Dermatology, University Medical Center of the Johannes Gutenberg University Langenbeck Street 1 D-55131 Mainz Germany; e Academy of Non-Profit Sciences in Erfurt Gotthardt Street 21 99084 Erfurt Germany; f Galenus GH AG Rain Street 7 6052 Hergiswil Switzerland; g Institute of Materials Science (ICMUV), Universitat de València C/Catedràtic José Beltrán 2 46980 Paterna - València Spain; h Division of Microbiology, Department of Pathology and Microbiology, Nihon University-School of Medicine 30-1 Oyaguchi-Kamicho Itabashi-Ku 173-2610 Tokyo Japan

## Abstract

Inorganic polyphosphate (polyP) is a metabolically relevant biopolymer involved in cellular energy storage and ATP-dependent tissue repair, including skin regeneration and wound healing. Here, a Good Manufacturing Practice (GMP)-compliant sodium polyphosphate formulation (Na-polyP-GMP) was developed and evaluated for regenerative applications, with a commercial polyP preparation used for comparison. Na-polyP-GMP exhibited enhanced physicochemical properties, characterized by a narrow, physiologically relevant chain length distribution and a fully amorphous structure, features associated with improved biological performance. *In vitro* studies using human keratinocytes and SaOS-2 cells indicated that Na-polyP-GMP supports ATP-dependent cell growth, differentiation, and microvilli formation, effects that were attenuated upon enzymatic ATP depletion, highlighting an energy-related mode of action. In exploratory, non-controlled proof-of-concept applications, topical administration of Na-polyP-GMP was associated with accelerated regeneration of a chronic venous ulcer and a therapy-resistant traumatic ulcer in two human patients, as well as with favorable healing of a persistent bacterially infected wound in a dog following failure of standard therapies. In addition, Na-polyP-GMP enhanced hair regeneration in diabetic mice and *ex vivo* human skin explants, accompanied by increased hair papillae density, stem cell proliferation, and vascularization. Together, these findings support the regenerative potential of Na-polyP-GMP and establish a foundation for its further exploration in wound healing and skin regeneration applications.

## Abstract 2

A GMP-compliant amorphous polyphosphate formulation acts as an energy-delivering biomaterial that promotes ATP-dependent skin regeneration, accelerating chronic wound healing and enhancing hair growth in human, veterinary and preclinical models.

## Graphical abstract

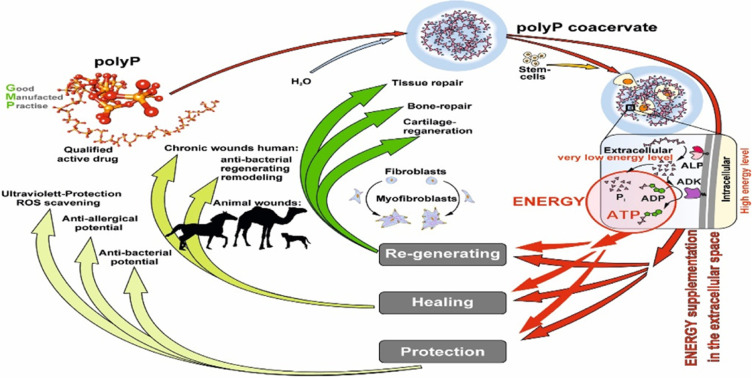

## 1. Introduction

Advanced skin care is essential for maintaining integrity and functional stability of the skin. As the body's largest organ, the skin serves as a protective barrier against external assaults. It requires a significant amount of energy to sustain its diverse functions. For instance, one crucial function—maintaining *trans*-epidermal water homeostasis (daily release of 300–400 mL)—demands an energy expenditure of 2000–3000 kcal day^−1^.^1^ More importantly, the skin is in a constant state of regeneration, renewing itself every 28 days. During this process, 30 000 to 40 000 dead skin cells are shed every minute.^2^ The metabolic energy required for these processes is primarily supplied in the form of ATP. A deficiency in ATP—such as in slow- or non-healing wounds caused by inadequate blood supply, particularly in ischemia—leads to reduced cellular energy availability.^3,4^

Unlike the intracellular space, where ATP levels range from 3–10 mM, the extracellular environment in mammalian tissues is extremely ATP-deficient, with concentrations of approximately 10 nM (discussed in ref. 5). Since mitochondria do not exist extracellularly, ATP cannot be synthesized *via* glycolysis coupled to oxidative phosphorylation. It has been postulated that ATP and ADP in the extracellular space are replenished directly by the blood platelets.^6^ Interestingly, along with these high-energy phosphates, platelet-derived growth factor (PDGF) and vascular endothelial growth factor (VEGF) are released from platelets.^6^ Given that extracellular ATP stimulates DNA synthesis^7^ and acts through an autocrine/paracrine circuit,^8^ researchers have proposed that ATP, in conjunction with these mitogens, could aid in treating chronic wounds—a hypothesis that has been experimentally supported.^4^

Chronic wounds, such as those caused by diabetes mellitus, pose a significant health risk with severe consequences for patients. These wounds represent a major economic burden for healthcare systems worldwide.^9–11^ In the U. S. alone, the prevalence of chronic wounds has risen over the past five years, reaching 16.4% in 2019.^12^ These wounds include surgical wounds, diabetic and infection-related wounds, pressure ulcers, and chronic ulcers. The estimated cost of chronic wound management stands at $126.86 billion in the U. S. and $15.97 billion in Germany.^13^ In Europe, these costs represent between 2 and 5% of total healthcare expenditure.^14^ Regardless of the specific type—diabetic wounds or venous ulcers due to chronic venous disease—the primary underlying cause is impaired blood flow, which results in reduced ATP supply to affected tissues.^15^ Additionally, metabolic imbalances, such as an increase in gluconeogenesis at the expense of glycolysis, further exacerbate disease progression.^16^ Cellular ATP levels are crucial for many functions, including the maintenance of insulin-secreting islet β-cells and insulin granule exocytosis, which play key roles in intermediary metabolism regulation.

The primary energy sensor in cells is AMP-activated protein kinase (AMPK), a multimeric, allosteric enzyme that regulates cellular metabolism. AMPK is a trimeric complex comprising a catalytic α-subunit, which is activated in response to an increased intracellular AMP/ATP ratio,^17^ as extensively discussed by the Solesio group.^18^ This group also highlighted the interaction of another high-energy physiological polymer, namely polyphosphate (polyP), with the activity of AMPK. When activated under nutrient-deficient conditions, AMPK enhances glucose uptake and lipid oxidation for energy production, while simultaneously downregulating energy-consuming processes such as glucose and fatty acid synthesis to restore energy balance.

Given the low levels of extracellular ATP^19,20^ and the high metabolic energy demands during tissue regeneration—even together with the complex building of the extracellular matrix^5,21^—researchers have sought an alternative extracellular energy source. This led to the discovery of polyP. PolyP is an energy-rich, inorganic, physiological linear polymer composed of three to several thousand orthophosphate (P_*i*_) units linked *via* high-energy phosphoanhydride bonds.^22–25^ Each phosphoanhydride bond in polyP stores approximately 30 kJ mol^−1^ of energy,^24^*i.e.* polyP stores significantly more energy than ATP, which contains only two such bonds. In addition to linear polyP, cyclic polyP molecules exist, which are transitionally formed during thermal synthesis of polyP, mainly trimetaphosphate consisting of three P_*i*_ units.^26^ Structurally, the polyP chains consist of tetrahedrally coordinated P_*i*_ units linked together *via* shared oxygen atoms. At neutral and alkaline pH, polyP is a strong polyanionic polymer with a negative charge on each internal P_*i*_ unit (dissociation constant p*K*_1_ = 2.2) and one to two negative charges on the terminal P_*i*_ residues (p*K*_1_ = 2.2 and p*K*_2_ = 7.2). The sodium salts of polyP (Na-polyP), with fewer than 100 P_*i*_ residues, are readily soluble in water. In addition to its function as an energy storage with the potential for ATP production, polyP is characterized by another exceptional property: at neutral pH, it forms a coacervate in the presence of divalent cations such as Ca^2+^ or Mg^2+^.^27,28^ This process involves a liquid–liquid phase separation, resulting in two liquid phases: a polymer-rich coacervate phase and a polymer-poor phase. The mechanism of polyP coacervate formation has been discussed.^28,29^ At alkaline pH values, especially at a superstoichiometric Ca : P ratio, polyP nanoparticles are formed with Ca^2+^ or other divalent cations, which can serve as storage forms of the polymer.^21^ Consequently, polyP exhibits a clear structure–function relationship; it can exist both as Ca-polyP nanoparticles (acting as depot form) and as free polyP or as polyP coacervate (biologically active form).

Principally, polyP is a stable molecule, despite its thermodynamic instability in aqueous solution. The kinetic stability of polyP at neutral or alkaline pH and room temperature is presumably due to the high density of negative charges in the polyP polyanion, which protects the molecule from hydrolytic attack by water.^30^ At acidic pH, hydrolysis occurs from the ends of the polymer.^31^

Physiologically, polyP is primarily stored in the blood platelets, which originate from megakaryocytes. Within the platelets, polyP is synthesized in the dense granules—organelles that resemble acidocalcisomes.^32^ While the enzymatic pathway of polyP synthesis in mammalian systems remains poorly understood, polyP is synthesized in bacteria by polyP kinases.^33,34^ In yeast, polyP is produced from ATP exported from the mitochondria *via* the Vtc complex, which resides in the acidocalcisomal membranes.^35^ Notably, the ATP concentration in the platelet-dense granules and acidocalcisomes is approximately 400 mM, compared to ≈150 mM in the mitochondria.^32^

PolyP is characterized by extraordinarily high biocompatibility and regenerative activity, particularly at the site of injury. This polymer plays numerous roles in mammalian cells, including serving as a metabolic energy reservoir,^36^ regulating bone mineralization,^37–39^ acting as a molecular chaperone,^40^ and responding to oxidative stress.^41^ Furthermore, polyP influences neurodegenerative diseases such as Parkinson's, Alzheimer's, and ALS^42,43^ and exerts protective effects in the respiratory tract epithelium.^44,45^ In particular, as also shown here, it was demonstrated that polyP-coacervate attracts stem cells, in which the polymer exerts its regenerative properties.^27,36^

This study presents for the first time a Good Manufacturing Practice (GMP)-compliant Na-polyP with a narrowly defined chain length distribution that exhibits superior cell growth and differentiation potential *in vitro*. GMP refers to a quality assurance system that is mandatory for the certified production of medicinal products for human use, which is controlled by the relevant government authorities. In this study, the effect of the sodium salt of GMP-compliant polyP (Na-polyP-GMP) on chronic wound healing is documented in two clinical cases in humans and the first chronic wound study in a dog. Additionally, given the mechanistic link between wound healing and hair growth,^46,47^ the anabolic effects polyP on hair regeneration are also explored. These findings highlight emerging role of polyP in regenerative medicine.

## 2. Experimental

### 2.1. Materials

The following products were purchased: McCoy's medium (Biochrom-Seromed, Berlin, Germany), 4-(2-hydroxyethyl)-1-piperazineethanesulfonic acid (HEPES; #H3375, Sigma-Aldrich, Taufkirchen, Germany), six-well plates from Orange Scientifique (Braine-l'Alleud, Belgium), and the MTT cell viability assay (Sigma-Aldrich).

### 2.2. Preparation of Na-polyP

Na-polyphosphate (Na-polyP) was synthesized in our laboratory using monosodium dihydrogen orthophosphate (NaH_2_PO_4_; S567545, Sigma-Aldrich) as a precursor. Polymerization was carried out in a furnace (Model AAF 3&7; Carbolite-Gero, Neuhausen, Germany) after preheating to 750 °C for 1 h, followed by maintaining this temperature for an additional h. An 180 mL glazed porcelain crucible from Haldenwanger (Roth, Karlsruhe; Germany) was used. At the end of the heating period, the crucible was removed from the furnace and the melt was poured onto a stainless steel plate at room temperature (“accelerated cooling”). The material was processed further after 30 min. Alternatively, the melt was left in the furnace until reaching room temperature (“slow cooling”; approximately 6 h). The preparation obtained after accelerated cooling was designated as Na-polyP-GMP. The original procedures were described by Partridge *et al.*^48^ and Ferrucci *et al.*^49^ The cooling process was monitored using an InfiRay ZH38 infrared camera (INFIRAY Thermokamera, Testo, Titisee-Neustadt, Germany). The permission for the Na-polyP-GMP has been granted. It is registered under the Certificate no: DE_RP 01_GMP 2023_0029.

In further studies, the melting process was carried out at furnace temperatures of 450 °C and 550 °C. Under these conditions, the formation of crystalline cyclic trimetaphosphate is favored,^26^ which was used for comparison. Also for comparison, commercial Na-polyP, labeled as Na-polyP-COM, was obtained from Chemische Fabrik Budenheim (Budenheim, Germany).

### 2.3. Characterization of Na-polyP-GMP *versus* Na-polyP-COM

#### 2.3.1. Cell culture experiments

##### SaOS-2 cells

Proliferation studies were conducted using human osteogenic sarcoma SaOS-2 cells,^50^ known for their high proliferation and differentiation potential.^51^ The cells were cultured as described by Wang *et al.*^52^ When necessary, cultures were buffered to pH 7.4 with 20 mM HEPES. Cell growth was quantified using the MTT cell viability assay,^53^ with ten parallel assays performed.

##### Keratinocyte assay system

For these studies, human epidermal keratinocytes (A549 cells; #102-05A, Sigma) were used. The cells were cultured in complete epidermal keratinocyte culture medium (#SCMK001, Sigma-Aldrich), supplemented with 5% fetal bovine serum (FBS; #F0850, Sigma) as described by Wang *et al.*^54^

##### Staining of actin stress fibers

The actin stress fibers in the myofibroblasts were reacted with anti-human-α smooth muscle actin (Clone 1A4), from Sigma #A2547. Tissue samples were fixed in paraformaldehyde and sliced.^5^ After deparaffination, the sections were stained with the antibodies against actin.^5^ Finally, the immunocomplexes were visualized in green with the FITC-labeled secondary antibody from ThermoFisher Scientific (Frankfurt, Germany). For visualization in red, the goat anti-Alexa Fluor-350 secondary antibodies, also from ThermoFisher, were applied. The specimens were inspected with a fluorescence microscope for green fluorescence: excitation source at 488 nm and emission at a wavelength of 535 nm (green) or 343/441 (red); as outlined.^55^

##### Scanning electron microscopy and energy-dispersive X-ray analysis

For scanning electron microscopic (SEM) analyses, a HITACHI SU 8000 (Hitachi High-Technologies Europe GmbH, Krefeld; Germany) was employed at low voltage (<1 kV; analysis of near-surface organic surfaces).^56^ The SEM microscope was coupled to an XFlash 5010 detector, an X-ray detector that allows simultaneous energy-dispersive X-ray (EDX)-based elemental analyses, which are at least semi-quantitative.

#### 2.3.2. Functional studies with human epidermal keratinocytes *in vitro*

##### Effect on cell growth

The impact of Na-polyP-COM and Na-polyP-GMP on cell growth was assessed using the MTT assay. In some experiments, keratinocytes were exposed to 60 µg mL^−1^ of the Na-polyP formulations. To evaluate the effect of ATP generated from polyP *via* the combined action of membrane-bound ALP and ADK,^57^ additional experiments were conducted using the ATP-metabolizing enzyme apyrase (200 U mg^−1^ protein; #A6535, Sigma). Assays were incubated with 10 U mL^−1^ apyrase to deplete ATP.^58^

##### Coacervation of Na-polyP in wound serous fluid

As previously reported,^59,60^ serous wound fluid contains the essential components for polyP phase transition into a coacervate, including Ca^2+^ (2 mM) and proteins (40 mg mL^−1^).^61^ To investigate whether Na-polyP-GMP and Na-polyP-COM form coacervates *in vivo*, an *in vitro* system with artificial wound exudate consisting of a mixture of inorganic salts and protein (BZ292; Biochemazone, Leduc, Alberta, Canada) was used. Samples were treated with 60 µg mL^−1^ of the Na-polyP formulations. The 24-well plates were loaded with 100 µL of wound exudate, followed by 100 µL of Na-polyP preparation (final polymer concentration: 60 µg mL^−1^). Keratinocytes were added at 3 × 10^3^ cells per mL. After 30 minutes of incubation, the coacervate layer was examined using electron microscopy after critical point drying.^62^

##### Morphological changes of keratinocytes grown on the coacervate layer

Keratinocytes were cultured in 24-well plates (1 mL per well) on the polyP coacervate layer for four days. The cell monolayers were subsequently analyzed for morphological changes, with a focus on microvilli formation.^63^

##### Determination of rheology of Na-polyP-GMP

The coacervates were prepared under identical concentration conditions with respect to Ca^2+^ and either Na-polyP-GMP or Na-polyP-COM. The Na-polyP stock solution (5 g in 100 mL) was subjected to a CaCl_2_·2H_2_O stock solution (14 g in 100 mL) at room temperature. After 30 min, the viscosity was determined with a Brookfield DV3T viscometer (Brookfield, Middleboro; MA) as described.^63^ The viscosity values are given in Pa·s.^64^

#### 2.3.3. Application forms of Na-polyP-GMP

##### Preparation of collagen-based mats

Compressed collagen mats containing Na-polyP-GMP (termed Na-polyP-GMP mats) were prepared as described by Schepler *et al.*^57^ Bovine collagen (type I) provided by Lando Biomaterials (Shenzhen, China) was used. During the sequential acid/neutral pH treatment, 10 mg mL^−1^ Na-polyP-GMP was incorporated. The material was layered onto a nylon mesh filter (100 µm) and subjected to a 20 g load for 20 minutes, compressing the mats to ≈1 mm thickness. Mats were stored in 70% ethanol until use.

##### Formulation of polyP-containing hydrogel

A solution of 200 mg/10 mL Na-polyP-GMP was prepared in purified water and filtered through a 0.2 µm PES membrane (#83.1826.001; Sarstedt, Nümbrecht, Germany). The hydrogel was prepared using hydroxyethyl cellulose (#4482-NATROSOL250 HX Pharm; Caelo, Hilden, Germany), 2 g in 20 g 1,2-propandiol (#2554; Caelo), 65 g purified water, and 10 g phosphate buffer (pH 6.5). After stirring and autoclaving at 121 °C, Na-polyP-GMP was added to a final concentration of 800 µg mL^−1^. The hydrogel was sterile-filtered and designated as polyP-GMP hydrogel.

##### Preparation of wetting solution/polyP hydrogel

A 120 mM phosphate buffer (pH 6.5) supplemented with 80 mM NaCl was enriched with 20% (v/v) propylene glycol (#398039, Sigma-Aldrich) for antimicrobial and antifungal properties.^65^ Finally, 300 µg g^−1^ Na-polyP-GMP was added, and the solution was sterile-filtered (Sterifix 0.2 µm; B. Braun, Melsungen, Germany).

### 2.4. Application of Na-polyP-GMP for healing of chronic wounds

#### 2.4.1 Human wounds

##### Ethics

All experiments were performed in accordance with the Guidelines of §37 of the Declaration of Helsinki,^66^ and Experiments were approved by the ethics committee at University Medical Center of the University Mainz. Informed consents were obtained from human participants of this study.

The first patient, an 83-year-old man, had suffered from a chronic venous ulcer on his right lower limb for more than 10 years. The second patient was an 82-year-old woman with a small but therapy-resistant traumatic ulcer on her left lateral malleolus that had been present for over nine months. The treatment of both wounds began with debridement (removal of necrotic or non-viable skin). The wound of the first patient was subsequently managed using the collagen-based Na-polyP-GMP mat and the Na-polyP-GMP hydrogel as indicated. The wound of the second patient was treated with the Na-polyP-GMP hydrogel only. The results of the corresponding vehicle control for this patient are also presented. The procedures followed previously described protocols^57,63^ and are detailed further in the “Results” section.

#### 2.4.2 Wound in a dog

##### Ethics

All animal procedures were performed in accordance with the Guidelines for Care and Use of the Laboratory Animals of University Medical Center of University Mainz and Experiments were approved by the Animal Ethics Committee of the Laboratory Animals of University Medical Center of University Mainz. The off-label application was based on scientific evidence indicating that animals suffering from severe pain and distress due to chronic wounds, such as the Shepherd dog in this case, benefit from polyP medication, experiencing at least some alleviation of symptoms. Written consent was obtained from the owner. The application adhered to EU standards as outlined in Directive 2010/63/EU.^67^ Both the dog owner and the veterinarian agreed.

On June 3, 2024, the 7-year-old dog self-inflicted a 3 × 4 cm wound on its right posterior hind limb. The wound was deep, exposing the sartorius muscle at its transition to the musculus biceps femoris, along with the associated tendons. Initially, the dog exhibited no signs of pain, possibly due to concurrent paralysis. However, dizziness and severe malaise were evident.

As an initial treatment, the antibiotic Prurivet (containing chloramphenicol and dexamethasone) was applied to the infected wound, following veterinary advice. Additionally, a natural, bioactive, and viscous skin medication, manuka honey ointment, was applied to create a protective barrier against superinfection and minimize scarring. Despite repeated applications of Prurivet combined with manuka honey ointment, along with compresses, cotton bandages, and cohesive dressings (last application: July 14), no significant healing was observed by July 21. Consequently, the veterinarian recommended the application of a hydrogel containing Na-polyP-GMP.

### 2.5. ATP pool determination

The protocol for the ATP pool determination was as previously described.^68^ Keratinocytes were cultivated, after reaching a density of 1 × 10^5^ with either Na-polyP-GMP or Na-polyP-COM at a concentration 60 µg mL^−1^. ATP was quantitated using an ATP-monitoring luminescence assay (No. LL-100-1; Kinshiro, Toyo; Japan). The ATP concentration is given in pmol per 10^6^ cells. The number of viable cells was determined with the Trypan Blue (#T8154; Sigma-Aldrich) exclusion test.^69^

### 2.6. Hair growth studies

#### 2.6.1. Experiments with mice

The *in vivo* experiments were conducted using genetically modified male diabetic mice, BKS.Cg-m + Leprdb/ + Leprdb (db/db), which exhibit delayed wound healing, aged 6 to 7 weeks upon arrival (Charles River, Calco, Italy).

##### Ethics

Ethical approval was obtained in accordance with Directive 2010/63/EU and national legislation governing the use of laboratory animals for scientific research and other purposes (Official Gazette 55/13). Additionally, an Institutional Committee on Animal Research Ethics (CARE-Zg) supervised all animal-related procedures to ensure compliance with animal welfare standards. The relevant permissions are documented in CAREZG_13-06-14_49 EP/2016 (SP-167-15 and SP-167-16) and KLASA: UP/I-322-01/15-01/108, URBROJ:525-10/0255-16-8, issued by the Ministry of Agriculture of the Republic of Croatia.

The experimental wound procedure followed an established protocol.^70,71^ The interscapular region was shaved, and a depilatory cream (Veet; Slough, UK) was applied to an area of approximately 10 mm. After disinfection under strict aseptic conditions, a full-thickness excisional wound (8 mm in diameter) was created along the midline using a sterile disposable biopsy punch, exposing the underlying fascia muscularis. Immediately afterward, pure Na-polyP-GMP powder was applied directly to the wound. The wound was then covered with a Tegaderm Wound dressing (3M, St. Paul, MN, USA), which remained in place until the study's conclusion at days 6 and 13. The mice were then humanely euthanized using an overdose of ketamine (Taj Pharmaceuticals, Newcastle, UK) and xylazine (KHBoddin, Hamburg, Germany), administered intraperitoneally.

#### 2.6.2. Experiments with human explants

##### Ethics

Human skin samples were collected in accordance with the rules of the declaration of Helsinki. In addition to these recommendations, the consent of the patients has been granted and the ethics committee of Rhineland–Palatinate gave its consent also (21.09.2022). No further medical ethics committee approval was required.

The cultivation of tissue samples followed an established protocol.^72^ The specimens were submerged in DMEM/F12 without phenol red (Gibco/Thermo Fisher, Dreieich, Germany), supplemented with 0.4% (v/v) bovine pituitary extract (#P1476, Sigma-Aldrich), 10 ng mL^−1^ epidermal growth factor (SRP3238, Sigma), 1 ng mL^−1^ basic fibroblast growth factor (3718-FB, R&D Systems, Minneapolis, MN, USA), 1 ng mL^−1^ vitamin E (#258024, Sigma-Aldrich), 1X insulin-transferrin-selenite (100-fold dilution; Gibco/Thermo Fisher), and 1% fetal calf serum (FCS) (Gibco/Thermo Fisher). Where indicated, the explant medium was supplemented with 200 μg mL^−1^ (w/v) Na-polyP-GMP (formulated in the hair tonic).

For histological examination, the tissue specimens were fixed in paraformaldehyde (#4760, Sigma) and embedded in paraffin wax (#03987, Sigma). The blocks were sectioned into ≈5 μm slices, mounted onto glass slides,^73^ and stained with hematoxylin and eosin (Mayer's hematoxylin, #MHS1, Sigma-Aldrich; eosin Y solution, #HT110280, Sigma-Aldrich) following established protocols.^74,75^ Microvessels were immunostained using anti-CD31 (PECAM-1) monoclonal antibodies (390, Gibco/Thermo Fisher) and recombinant Ki-67 rabbit monoclonal antibodies (SP6, Thermo Fisher). Immunocomplexes were visualized using an ALP-labeled anti-mouse secondary antibody (GtxMu-004-EALP, Dianova, Hamburg) and developed with nitro blue tetrazolium chloride (NBT, #N5514, Sigma) and 5-bromo-4-chloro-3-indolyl phosphate (BCIP, #B6149, Sigma).

#### 2.6.3. Hair tonic formula

A base solution of 70% (w/v) purified water and 10% (w/v) Aloe vera leaf extract^76^ was prepared to provide moisturizing and soothing properties. Additionally, 5% (w/v) invertin (#1.04738, E. Merck, Darmstadt, Germany) was included as a softening agent.^77^ A few drops of colorant and fragrance (prized vanilla/white musk) were added for aesthetic appeal. Finally, 600 μg mL^−1^ (w/v) of Na-polyP-GMP was incorporated, with the final pH adjusted to 6.5 (with phosphate buffer).

### 2.7. Analytical methods

Analysis of polyP chain length: the size of the polyP preparations was determined by electrophoresis on high percentage urea polyacrylamide gels (16.5% polycrylamide/7 M urea) using polyP standards of defined chain length, as described.^37^ The gels were stained with toluidine blue.

SEM analysis: scanning electron microscopy (SEM) was performed using a HITACHI SU 8000 electron microscope (Krefeld, Germany). Samples were placed on an aluminum grid and mounted on a SEM holder.

ESEM observations: environmental scanning electron microscopy (ESEM) was conducted using a Philips microscope (Eindhoven, Netherlands). During the procedure, cells shrank by approximately 35% of their volume.

Light microscopy: specimens were analyzed using a VHX-600 Digital Microscope (Keyence, Neu-Isenburg, Germany) equipped with Nomarski optics, or with a fluorescence microscope.

### 2.8. Statistical analysis

Statistical analyses were performed using Student's *t*-test. Data represent averages from ten independent experiments. Statistical significance was set at *p* < 0.01 (*). GraphPad Prism 7.0 software (GraphPad, La Jolla, CA, USA) was used for data analysis.

In one series of experiments the correlation coefficient of significance was *p* < 0.05.

## 3. Results

### 3.1. Advanced preparation of Na-polyP (Na-polyP-GMP)

#### 3.1.1. Thermal synthesis of the GMP-compliant Na-polyP

For use in humans, a GMP-compliant polyP preparation should have well-defined properties, such as a specific size or size distribution, as has been shown for Na-polyP-GMP. In addition, it is considered to be advantageous when the material is present in the amorphous phase.^21,36,78^ For the thermal condensation of NaH_2_PO_4_ to Na-polyP, we followed this protocol: preheating the furnace to 750 °C for 1 h, adding NaH_2_PO_4_, and maintaining condensation again at 750 °C for another 1 h. The post-condensation period lasted 30 minutes, during which the temperature gradually cooled from 750 °C to room temperature. A key issue to address was the prevention of the decrease in pH (acidification) that typically occurs when commercial Na-polyP dissolves in medium/serum. It was observed that after adding 10 mg ml^−1^ Na-polyP-COM to water, a sharp drop in pH to about 2 was observed, while the pH remained constant during the dissolution of Na-polyP-GMP. This pH drop caused by Na-polyP-COM could be due in the degree of dissociation of the Na-polyP salts with increasing chain length of the polymer,^79,80^ or to hydrolytic cleavage at branching points,^81^ which may be present in commercial Na-polyP preparations and lead to an increased number of acidic hydroxy groups at the ends of the resulting oligophosphate chains.

**Fig. 1:**
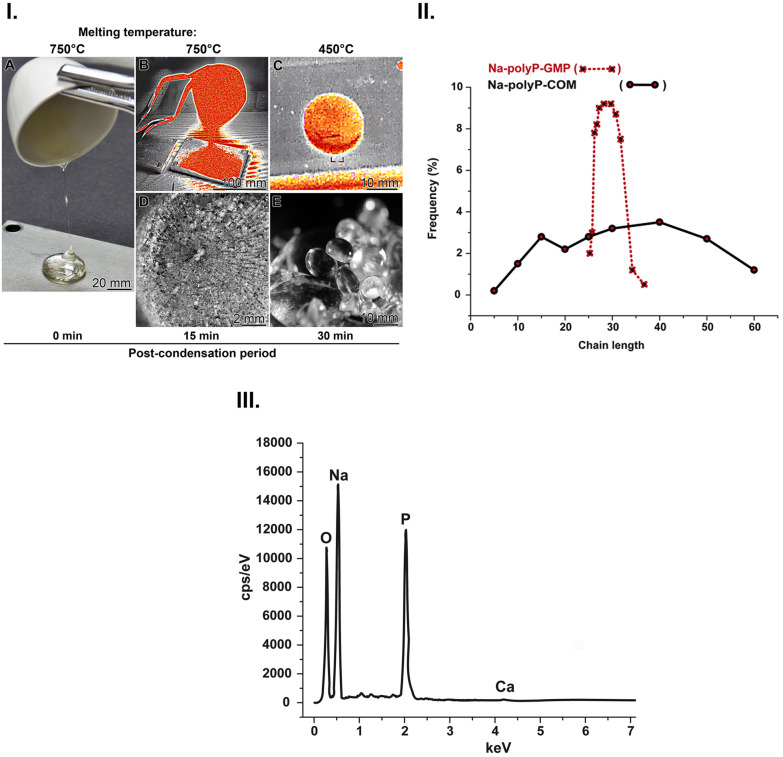
Synthesis of Na-polyP from NaH_2_PO_4_ monomers. The polymer was prepared at 750 °C using a controlled polycondensation process, followed by accelerated cooling (B to C). (I.) (A) Pouring the melt from the crucible (83 mm upper diameter and 55 mm height). Two time points were chosen to demonstrate polyP lens formation; after a post-condensation period of (D) 15 min and (E) 30 min. Perfectly convex lenses are formed after 30 min. At time point 15 min, the lenses are still fused together. The images (B) and (C) were taken with an infrared camera; the square in (C) is the camera focus. (II.) The sharp distribution of polyP chain lengths (given in P_*i*_ units) of the Na-polyP-GMP polymer (red) in contrast to the broad size distribution of Na-polyP-COM (black). (III.) EDX spectrum for the Na-polyP-GMP formulation showed the prominent element peaks for Na, O, and P; the weak Ca peak originates from the crucible material.

The applied time regimen was successful. As described in the following section, the Na-polyP-GMP preparation was perfectly amorphous, a property expected to be advantageous for a bioactive material (see above). After polycondensation at 750 °C, the melt was viscous when poured from the crucible (Fig. 1(I-A and I-B)). During the post-condensation/cooling phase, Na-polyP lenses evolved from coarse granules (Fig. 1(I-D); 450 °C) to perfectly convex lenses (Fig. 1(I-E)) within 15 to 30 minutes; Fig. 1(I-C) shows the infrared camera image of a still warm lens.

#### 3.1.2. Physico-chemical characterization of the advanced Na-polyP preparation

##### PolyP size analysis

The Na-polyP-GMP preparation displayed a notably narrow chain length distribution of 26 to 37 P_*i*_ units (Fig. 1(II)), peaking at 30 P_*i*_ units. In contrast, the length distribution of the commercially available Na-polyP-COM product ranged from 5 to 60 P_*i*_ units. The Na-polyP-GMP size range thus meets the criteria for chemotactic activity^82^ and is close to maximal induction of human fibroblast differentiation into the myofibroblast phenotype^83^ without exhibiting negative effects due to induction of coagulation or release of bradykinin expression shown by the longer-chain polyP molecules.^84–86^

##### EDX spectral analysis

The element distribution of the Na-polyP-GMP was assessed by EDX analysis (Fig. 1(III)). The spectrum of Na-polyP-GMP shows only three main peaks corresponding to O, Na, and P, with no other contaminating elements. In addition, a small Ca peak (from the porcelain crucible) is visible. The Na : O ratio is slightly higher than the stoichiometrically expected value, since the termini of the polyP chain can bind two Na^+^ ions.^87^ The P : O ratio is close to 1 : 2 (selection of five points on the glass surface).

##### Microscopic analysis

**Fig. 2:**
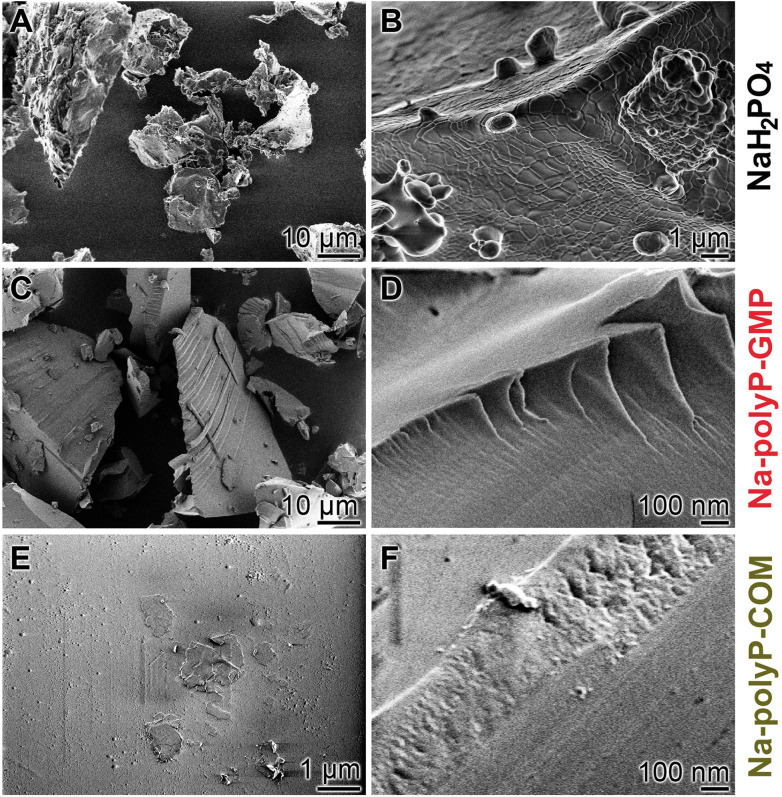
Morphology of solid Na-polyP deposits and its NaH_2_PO_4_ starting material; SEM. (A and B) Aspects of monomeric sodium phosphate. (C and D) Images of Na-polyP-GMP blocks. (E and F) The Na-polyP-COM samples show layers based on a granulated bottom (granulated particles).

In contrast to the NaH_2_PO_4_ sample, which exhibited (at lower magnification) a brittle morphology, with flowing planes adorned with drop-shaped convexities (Fig. 2A and B), the polymeric Na-polyP-GMP blocks had a perfectly smooth appearance with interleaving layers (Fig. 2C and D). Na-polyP-COM samples also displayed plane surfaces but were layered over a granulated base (Fig. 2E and F).

##### XRD analysis

**Fig. 3:**
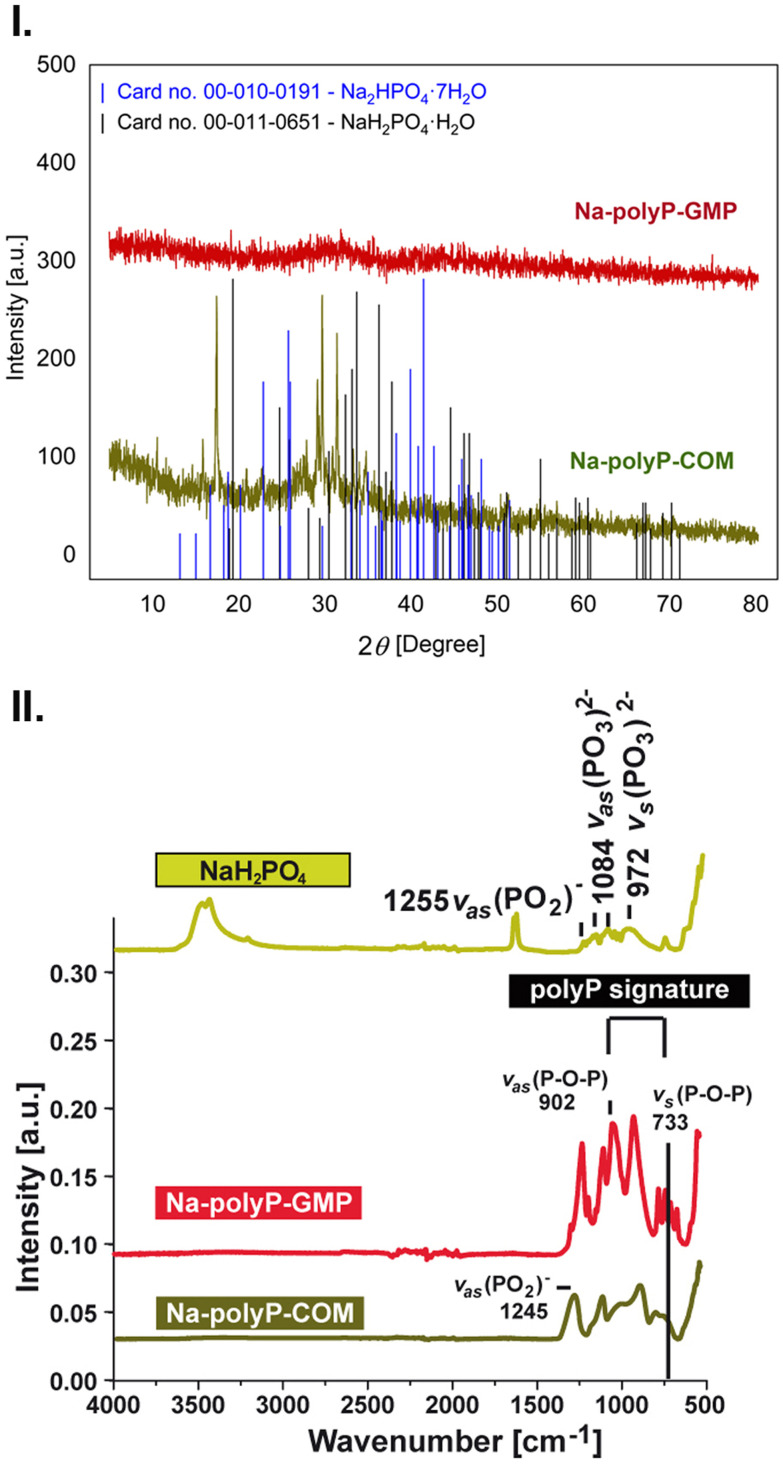
(I.) XRD analyses of Na-polyP-GMP compared to Na-polyP-COM. The commercial product shows some crystalline signals. (II.) FTIR analysis. In comparison to the NaH_2_PO_4_ starting material, the two Na-polyP preparations Na-polyP-GMP and Na-polyP-COM show the two characteristic polyP signatures at *v*_as_ and *v*_s_ for P–O–P.

NaH_2_PO_4_, used as the starting material for Na-polyP condensation, is crystalline. During heating, the crystals transformed into an amorphous solid, as observed in the Na-polyP-GMP polymer (Fig. 3(I)). In comparison, the commercial Na-polyP contained crystalline signals.^88^ The XRD reflections of the Na-polyP-COM sample cannot be assigned to a single crystalline phase and instead appear to represent a mixture of different phases, most likely sodium hydrogen phosphate hydrate phases, such as NaH_2_PO_4_·H_2_O (PDF card no. 00-011-0651) or Na_2_HPO_4_·7H_2_O (PDF card no. 00-010-0191).

##### FTIR spectral analysis

FTIR analysis of the monomeric sodium phosphate starting material for Na-polyP preparation revealed characteristic signals at *ν*_as_ 1255 for (PO_2_)^−^, *ν*_as_ 1084 for (PO_3_)^2−^, and *ν*_s_ 972 for (PO_3_)^2−^ (Fig. 3(II)). Both Na-polyP-GMP and Na-polyP-COM preparations displayed polyP signatures at *ν*_as_ 902 (P–O–P) and *ν*_s_ 733 (P–O–P).

##### Rheology

The rheological results show that the coacervate formed by Ca^2+^ and Na-polyP-COM has a value of 12.5 ± 1.5 Pa·s, reflecting the long-chain Na-polyP nature of this polymer.^64^ In contrast, the value for the Ca^2+^ coacervate of Na-polyP-GMP is 38 ± 53 Pa·s, consistent with the optimal range for a skin hydrogel and more physiological characteristics.^89^ The lower viscosity of the coacervate formed by Na-polyP-COM could also be due to a higher content of phosphate monomers or very short P_*i*_ oligomers in this preparation. These data confirm that Na-polyP-GMP is superior to Na-polyP-COM in terms of bio-compatibility and applicability as a hydrogel.

#### 3.1.3. *In vitro* characterization of the advanced Na-polyP preparation

##### Cell growth

**Fig. 4:**
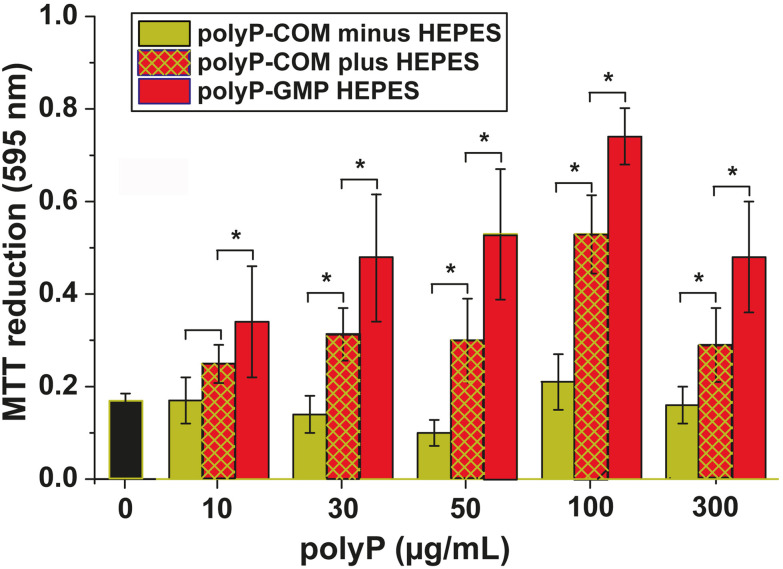
Effect of the two Na-polyP samples, Na-polyP-COM and Na-polyP-GMP, on growth/viability of SaOS-2 cells. In the absence of HEPES, the Na-polyP-COM samples are even inhibitory at pH values below 50 µg mL^−1^ compared to control (0 µg mL^−1^), whereas in the presence of HEPES, a significant increase in viability is observed. In contrast, Na-polyP-GMP is stimulatory to the cells even in the absence of HEPES. Ten parallel experiments were performed. The data are means ± SD (*p < 0.01).

The effect of Na-polyP samples on cell growth was assessed using SaOS-2 cells, known for their high proliferation and differentiation potential. Growth kinetics (Fig. 4) with the Na-polyP-GMP and Na-polyP-COM samples were examined using the MTT (MTT tetrazolium reagent to measure the number of viable cells in culture) cell viability assay. The data showed that with Na-polyP-COM, the growth rate only increased slightly (by ≈25%) within the three-day incubation period at concentrations above 100 µg mL^−1^. However, when the culture system was buffered with 20 mM HEPES at pH 7.4, viability significantly increased by 48% at 10 µg mL^−1^, and a 2.6-fold increase was observed at 100 µg mL^−1^.

In contrast, Na-polyP-GMP exposure led to significant growth augmentation at all concentrations above 10 µg mL^−1^, even without HEPES buffering (Fig. 4). Parallel studies revealed that in medium/serum supplemented with 10 mg mL^−1^ Na-polyP-COM, pH dropped by 2.5 units, whereas in Na-polyP-GMP, pH remained stable at 6.5 (data not shown). Consequently, in previous studies, assays were always buffered with 20 mM HEPES to prevent pH-induced cellular stress.

### 3.2. Structure–activity relationships

**Fig. 5:**
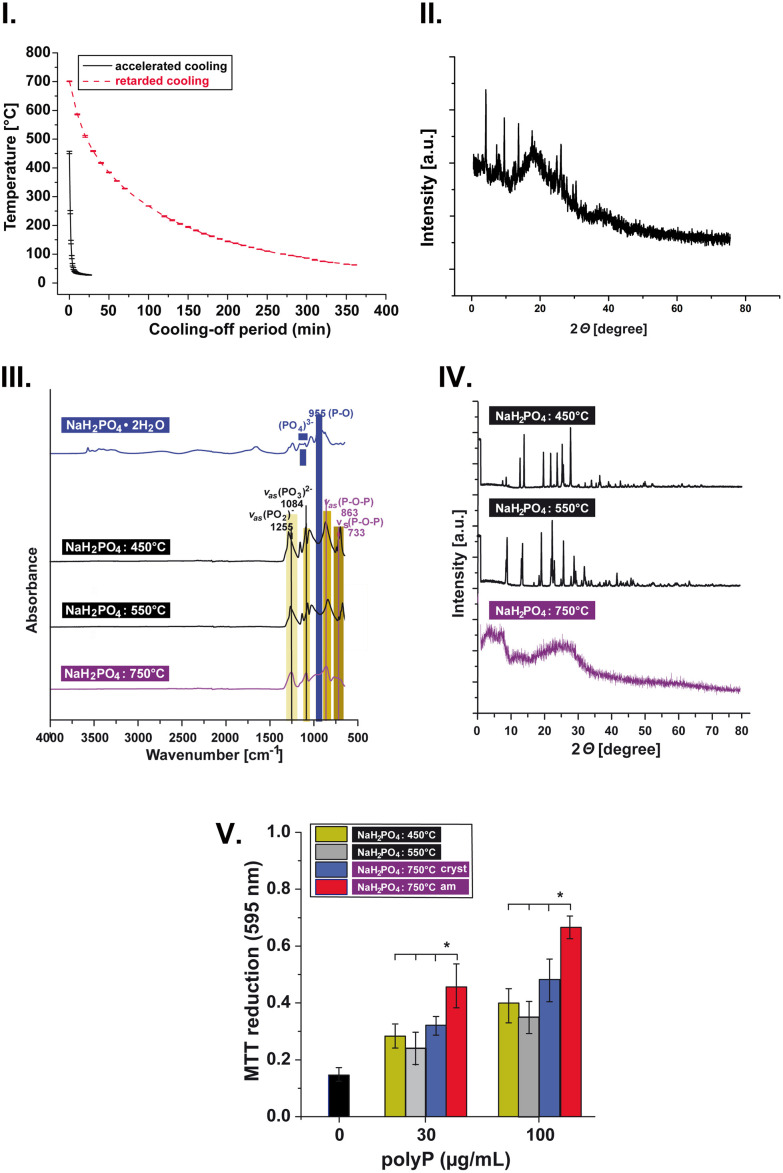
Structure–property relationships of Na-polyP. (I) Temperature profile of the Na-polyP melt formed at 750 °C after pouring onto a stainless steel plate (“accelerated cooling”) of when left in the furnace to reach room temperature (“slow cooling”). (II) XRD analysis of the Na-polyP sample obtained after slow cooling of the material obtained by heating of NaH_2_PO_4_ at 750 °C for 1 h. (III) FTIR spectra of the materials formed after melting NaH_2_PO_4_ and at 450 °C and 550 °C, compared to the spectrum of Na-polyP-GMP obtained at 750 °C. (IV) Diffractograms of the crystalline polyP materials obtained from NaH_2_PO_4_ at 450 °C and 550 °C compared to the amorphous Na-polyP-GMP product obtained at 750 °C. (V) Different efficiencies of various preparations obtained from NaH_2_PO_4_ at 450 °C, 550 °C, and 750 °C, either amorphous (am) or partially crystalline (cryst) on growth/viability of SaOS-2 cells. Means ± SD (*p < 0.01; *n* = 8).

It has been suggested that a material must be amorphous to exhibit biological activity. As discussed in previous studies, only amorphous, but not crystalline, biomaterials are thought to possess the potential for biological activity.^21,36,78^ Besides the phase of the Na-polyP material (amorphous *versus* crystalline), the size and type of polymer (linear or cyclic) can also be crucial for the biological performance of the material. To demonstrate the importance of the amorphous character of the preparation, Na-polyP was prepared from NaH_2_PO_4_ under the same conditions as described for Na-polyP-GMP (heating at 750 °C for 1 h), except that the resulting melt was not subjected to accelerated cooling to room temperature. Fig. 5(I) shows the temperature profile of the Na-polyP melt formed at 750 °C and cooled to room temperature in the furnace (“slow cooling”), compared to the profile after pouring onto a stainless steel plate (“accelerated cooling”). XRD analysis of the obtained material showed that Na-polyP exhibits a significant degree of crystallinity after slow cooling (Fig. 5(II)). Studies of the effect of this preparation on SaOS-2 cells showed that the Na-polyP phase has a significant influence on its biological activity. The partially crystalline Na-polyP was significantly less effective in simulating growth/viability of the cells than the purely amorphous Na-polyP-GMP obtained after delayed cooling (Fig. 5(V)).

To further demonstrate the relationship between the phase, type and chain length of the Na-polyP material with respect to biological performance, cyclic Na-polyP (cyclic Na-trimetaphosphate; (NaPO_3_)_3_) was prepared by a thermal process as described in literature.^81^ It is known that the heating process is accompanied by the stepwise removal of water, first the crystal water, followed by the removal of water through the condensation reactions during the formation of sodium pyrophosphate, cyclic sodium trimetaphosphate and, finally, at temperatures above 620 °C, a Na-polyP melt. Fig. 5(III) shows the FTIR spectra of the materials obtained from NaH_2_PO_4_ at 450 °C and 550 °C, compared to the spectrum of Na-polyP-GMP (750 °C). In XRD analysis, the cyclic Na-trimetaphosphate (heating at 550 °C) showed a diffractogram typical for a crystalline material, while the Na-polyP obtained at 750 °C and accelerated cooling was amorphous (Fig. 5(IV)). The crystallinity of the material obtained at 450 °C was somewhat lower. Both the crystalline Na-trimetaphosphate and the partially crystalline Na-polyP preparation obtained at 750 °C followed by slow cooling showed a markedly reduced stimulatory effect on growth/viability in the SaOS-2 cell system compared to the fully amorphous Na-polyP-GMP. This underscores the importance of the amorphous character, size, and type of polyP for its biological activity (Fig. 5(V)).

### 3.3. Application of Na-polyP-GMP for the treatment of chronic wounds in humans and animals

The application was conducted as an exploratory, controlled proof-of-concept case to evaluate the feasibility and biological response to Na-polyP-GMP treatment under supervision by a physician.

In general, the situation and course in human and animal chronic wounds are very similar^90^ and proceed in the sequence hemostasis, inflammation, proliferation, and remodeling. However, there is one process that is different between human and animal chronic wounds, which lies in the differentiation state of myofibroblasts involved in new tissue formation during the proliferation/remodeling phase of wound repair.^91^ Chronic wounds in humans are caused by exhaustion, failure or dysregulation of myofibroblast differentiation, while chronic wounds in dogs are caused by overshooting differentiation of fibroblasts to myofibroblasts.^92^ In both cases, the result is a delay in cutaneous wound healing and a retardation of granulation and lower contraction during the healing process. Furthermore, the restoration of the subcutis is slow, as in wound healing, due to the slowed removal of necrotic/apoptotic fragments of the decaying subcutis tissue.^93,94^ These aspects are important because in previous publications^5,57^ we suggested that the differentiation of fibroblasts into myofibroblasts is triggered by ATP generated from polyP during the sequential enzymatic degradation of the polymer.^5^

Especially due to the outlined differences in the course of wound healing with regard to the role of the myofibroblasts, we decided, continuing our previous studies (for a recent summary, see ref. 5), to investigate the efficiency of the polyP-enriched wound healing formulations in three proof-of-concept studies, two on chronic human wounds and one on a chronic animal wound.

**Fig. 6:**
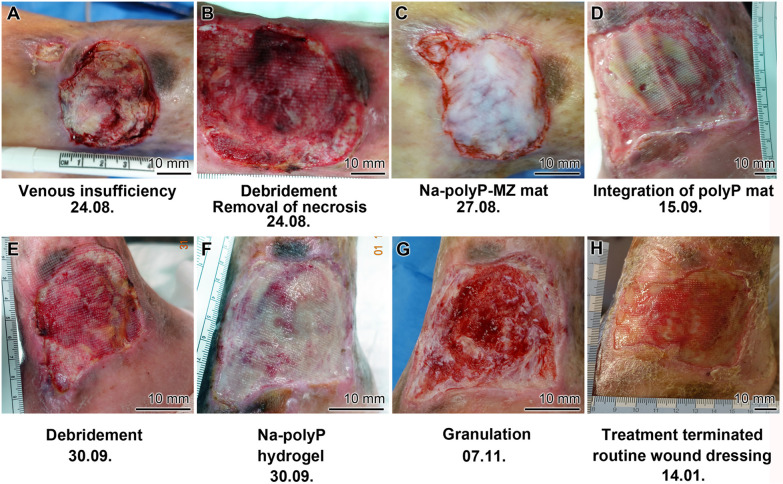
Steps during healing of the first chronic human wound, a chronic venous ulcer on the right lower limb. (A) Initial appearance. (B) Removal of necrotic tissue – debridement. (C) Coverage with a collagen mat supplemented with Na-polyP-GMP. (D) Integration of the polyP mat into the granulation tissue. (E) Conditioning of the wound. (F) Application of a Na-polyP-GMP supplemented hydrogel. (G) Granulation process. (H) Termination after a five months treatment with Na-polyP-GMP.

The new Na-polyP-GMP formulations were applied to the human wounds following standard guidelines.^95^ The first patient, an 83-year-old man, had suffered for over 10 years from a therapy resistant chronic venous ulcer on the right lower limb near the medial ankle (Fig. 6A). The wound bed was necrotic, containing sloughy deposits and devitalized skin in the surrounding tissue. Treatment began with debridement, involving surgical removal of necrotic tissue and gentle low-frequency ultrasound application (Fig. 6B). Since the wound was deep, a collagen-based mat was applied three days later (Fig. 6C), taking care to moisten it every 2–3 days with a Na-polyP-GMP solution.

After three weeks, granulation tissue emerged from the wound margins, integrating partially with the collagen-based mat (Fig. 6D). After superficial cleaning and conditioning of the wound on the integrated mat (Fig. 6E), the wound was covered with Na-polyP-GMP hydrogel (Fig. 6F). Over the next month, vigorous granulation tissue formation facilitated healing (Fig. 6G), allowing standard wound care to resume after ten additional weeks (Fig. 6H). The wound stabilized further over the following three weeks. A split-thick skin transplant was rejected. Nevertheless, the patient could be released home after 5 months.

**Fig. 7:**
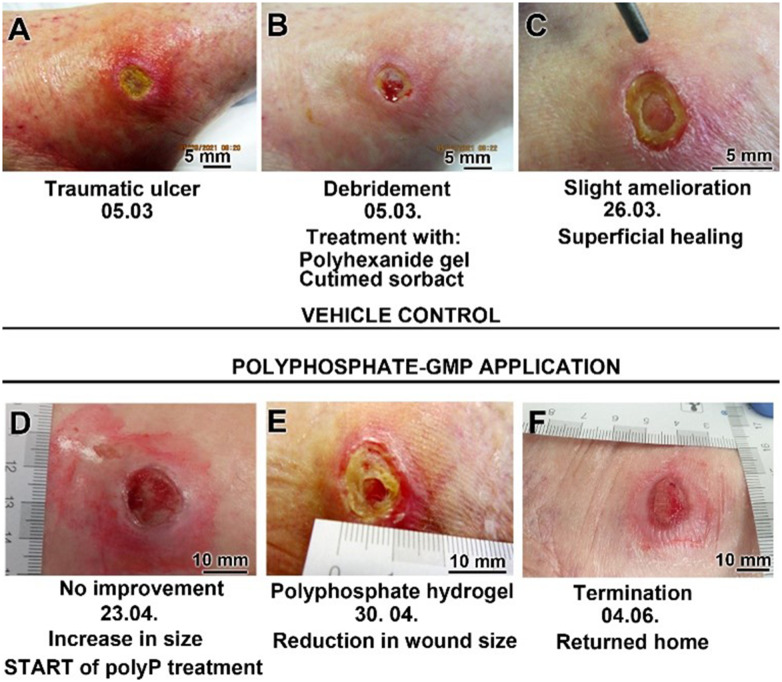
Healing of the second chronic human wound, a therapy-resistant traumatic ulcer on the left lateral malleolus. (A) Initial aspect. (B) Start of standard therapy with debridement and hydroxyethyl cellulose gel (vehicle control), supplemented with polyhexanide, and wound dressing. (C) Slight progress after 3 weeks, (D) however, no improvement after 7 weeks. Subsequently, start of treatment with Na-polyP-GMP using the same hydrogel base (hydroxyethyl cellulose gel). (E) Granulation tissue formation and wound size reduction. (F) Termination of Na-polyP-GMP treatment and discharge of the patient after six weeks.

The second patient, an 82 old woman, had been suffering from a small therapy-resistant traumatic ulcer on her left lateral malleolus since 6/2020. Peripheral arteriosclerosis and chronic venous insufficiency were ruled out. Despite various standard therapies with different wound dressings, including alginate dressings, gauze pads, and superabsorbent dressings, the ulcer did not heal. The patient presented in March 2021 with redness, pain, and signs of inflammation (Fig. 7A). The initial finding was an ulceration of the left lateral malleolus. Notable features included tissue loss, sloughy tissue, and strewn crater-like openings.

Following hospital admission and subsequent debridement (Fig. 7B), the patient received standard therapy with hydroxyethylcellulose gel (vehicle control) with polyhexanide (antibacterial polymer) and Cutimed sorbact as a wound dressing. After three weeks, superficial healing was observed (Fig. 7C). However, at the follow-up examination four weeks later, the ulcer appeared more superficial and had significantly enlarged without any substantial improvement (Fig. 7D). Furthermore, extensive redness with sharp edges had developed, suggesting an allergic contact reaction to the standard wound dressing. The patient also complained of pain and difficulty walking.

In this situation, it was ethically unacceptable to withhold from the patient the Na-polyP-GMP, which had previously been proven to promote healing.^57,63^ The patient was informed about this off-label approach for therapy-resistant cases and gave her consent.

After just one week, a reduction in wound size and the formation of granulation tissue were visible (Fig. 7E). Six weeks later, after changing the Na-polyP-GMP gel (1 to 2 mL) every other day, the ulcer showed well-vascularized tissue and a thin epithelial layer, indicating successful wound closure (Fig. 7F). The patient was able to be discharged home.

These controlled studies demonstrate that Na-polyP-GMP is an effective component for the successful treatment of a chronic wound after previous standard therapies had failed.

### 3.4. More efficient ATP release in keratinocyte cultures in the presence of Na-polyP-GMP

Keratinocyte cultures were used to determine the ATP release from the cells by using the established ATP-monitoring luminescence assay, as outlined under Methods. The determined concentrations refer to 10^6^ cells.

**Fig. 8:**
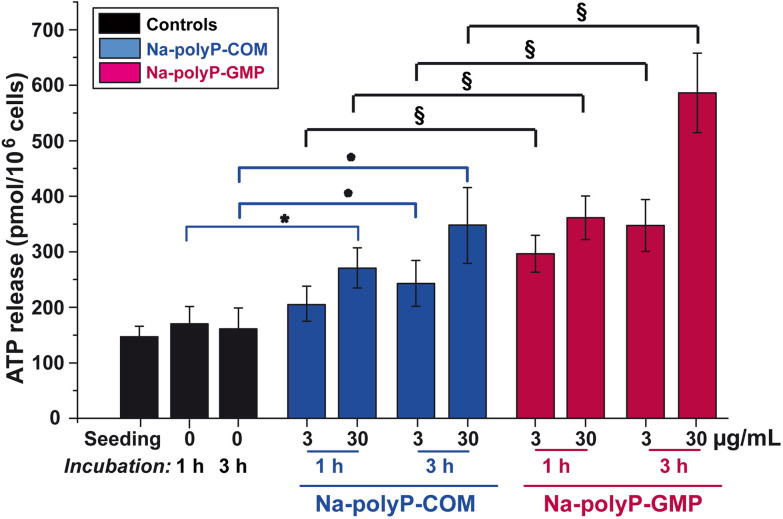
Effect of the two Na-polyP formulations, Na-polyP-COM and Na-polyP-GMP, on ATP production and release. The significance level was set at *p* < 0.05. Significant differences in the values of both Na-polyP formulations compared to the control after incubation for 1 h or 3 h were marked with (*) for Na-polyP-COM and with (§) for Na-polyP-GMP.

The results revealed that the ATP level in the control culture medium did not change significantly during the incubation period of up to 3 h. Compared with this baseline value, the ATP concentration in the medium supplemented with Na-polyP-COM increased significantly by 49% at a concentration of 3 µg mL^−1^ and by as much as 114% at 30 µg mL^−1^ (Fig. 8). However, when the keratinocyte cultures were incubated with Na-polyP-GMP under otherwise identical conditions, ATP release after 3 h was strongly enhanced (Fig. 8); at 3 µg mL^−1^, the release was 1.42-times higher, and at 30 µg mL^−1^ it was even twice as high as with Na-polyP-COM (Fig. 8).

As summarized in ref. 5, alkaline phosphatase (ALP) is the first enzyme involved in enzymatically controlled ATP synthesis from polyP, followed by the subsequent adenylate kinase (ADK) reaction. Like ADK, this enzyme is ubiquitous, not only in serum but also on cell surfaces.^96–98^ Due to this ubiquity, kinetic degradation analysis is less significant. Nevertheless, it can be deduced that increased ATP release occurs during the first 3 hours of incubation with Na-polyP-GMP, reflecting a significant upregulation of both enzymes compared to Na-polyP-COM (Fig. 8).

### 3.5. Na-polyP-GMP stimulates hair growth

Wound healing is closely linked to hair follicle regeneration.^45^ Both processes, wound healing and hair growth, have in common that they rely on epithelial stem cells. The formation of new hair follicles after injury—also known as wound-induced hair follicle neogenesis^99^—involves various signaling pathways, such as the Wnt/β-catenin pathway,^100^ and is observed in mammals, including mice and humans. Tissue regeneration in wound healing is particularly promoted during the anagen phase of the hair cycle.^46^ The stem cells, which also accumulate around regenerating skin cells, migrate from the follicle and induce re-epithelialization of skin wounds and the hair shaft.^101,102^ These cells are the drivers for the regeneration process. We therefore also investigated – comparatively – the effect of Na-polyP-GMP on hair growth.

#### 3.5.1 Diabetic mice

**Fig. 9:**
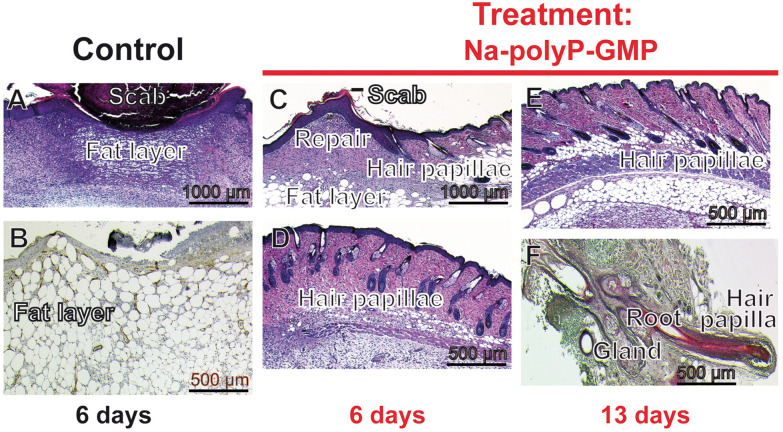
Growth of hairs adjacent to wounds in diabetic mice. Concentric wounds (8 mm in diameter) were punched into the skin of male diabetic mice above the muscular fascia. The shaved areas before wound setting were ≈10 mm. The wounds remained either untreated (A and B) or were treated/moistened with Na-polyP-GMP for 6 to 13 days (C to F) as described in the Experimental section. (A and B) In untreated control animals, a bulgy wound scab covered the defect from the first day after the wound was punched. After 6 days, a large fat layer formed in the defect area (B; Nomarski optics). (C to F) In contrast, treatment of the wounds with Na-polyP-GMP caused an intensive tissue repair and sealing-in of the scab. The wound defect becomes filled with granulation tissue (C and D). Already at day 6, hair papillae are lined up at the epidermis-dermis border. Only a restricted fat layer is seen. (E) During the following regeneration period (13 days), the density of the hair papillae and their size increased. (F) Occasionally, prolonged hair growth is seen, and hair roots and sebaceous glands can be recognized.

The effect of Na-polyP-GMP on wound healing and hair regrowth was investigated in a study on diabetic male mice showing delayed wound healing (Fig. 9). Eight-millimeter circular wounds were created above the muscular fascia. Control wounds were left untreated (Fig. 9A and B), while treated wounds were covered with ≈10 mg of Na-polyP-GMP for 6 to 13 days (Fig. 9C–F). The results of additional controls have been documented in a previous report.^71^

Histological analysis showed that untreated wounds developed scabs from day 1 (Fig. 9A), with fat layers still bordering the site on day 6 (Fig. 9B). In contrast, Na-polyP-GMP-treated wounds exhibited vigorous regeneration, forming ≈10 mm thick granulation tissue within six days (Fig. 9C). Inspection revealed early hair papillae formation at the epidermis-dermis border (≈100 µm from the epidermal surface), which became more distinct at higher magnification (Fig. 9D). Over the following week, hair papilla density increased (Fig. 9E), with visible hair elongation and sebaceous gland formation (Fig. 9F). These results confirm again the stimulatory activity of Na-polyP-GMP in the hair growth model.

#### 3.5.2 Human scalp hair growth

A more detailed analysis was performed using human hair explants. The hair growth center resides in follicular growth zones.^103^ The follicles are dynamic mini-organs characterized by intense growth and differentiation from ectodermal hair follicle stem cells.^104^ As outlined in the Experimental section, the hair explants were cultured *ex vivo* in the absence or presence of Na-polyP-GMP.

**Fig. 10:**
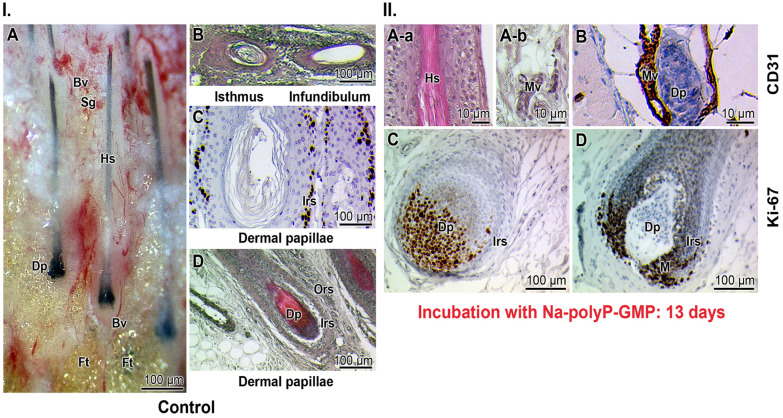
Explant cultures of hair/skin tissue in dependence of Na-polyP-GMP exposure. (I.) Histology of the control specimens, not incubated with polyP. (A; hematoxylin/eosin) Longitudinal section of the dermal papilla (Dp) along the hair sheath (Hs). The **s**ebaceous gland (Sg) region is highly vascularized (Bv, blood vessel), as is that around the dental papillae. In the images, fat cells/tissue are highlighted due to their light reflection (Ft). (B) Cross-section of the isthmus and the infundibulum region of the growing hair. (C and D; hematoxylin/eosin; immunostaining with anti-CD31) Dermal papilla, which houses the stem cells and is surrounded by blood vessels. The inner root sheath (Irs) and the outer root sheath (Ors) are indicated. (II. hematoxylin/eosin) Onset of vascularization and proliferation of endothelial cells after exposure of the explants to Na-polyP-GMP; the incubation period was 13 days. (A-a) Microvessels are formed around the hair sheath. (A-b; CD31 antibodies) Immune histology analyses with CD31 antibodies reveal a strong staining of the microvessels (MV). (B; hematoxylin/eosin/CD31) Around the dermal papilla, the microvessel formation is strong. (C and D; Ki-67 antibody reaction) Immune reactions with Ki-67 indicate a strong proliferation activity of the respective cells. The antibody reacts strongly with the cells within the inner root sheath and its matrix (M) region. The reaction is particularly strong during the invagination process (from C to D), during which the globular papilla is integrated into the dermal papilla.

The culture medium for the explants was enriched with bovine pituitary extract and two growth factors, as listed in the Experimental section. The control medium was not supplemented with polyP (Fig. 10(IA and I-B)), while the test specimens were exposed to 200 μg mL^−1^ (w/v; final) Na-polyP-GMP. The tissue response in the explants was documented by histology, including immunohistology.

In the control samples, the explant displays the dermal hair papilla along with the elongated hair sheath and associated sebaceous glands (Fig. 10(I-A)). Further distally, in cross-sections, the isthmus and infundibulum regions of the growing hair are visible (Fig. 10(I-B)). The axial section reveals the upper, permanent or fixed segment, and the lower segment with its growing mobile zone.^105^ The upper segment, also known as the hair shaft, eventually protrudes from the skin but does not contribute to the formation of new hair. In contrast, the hair bulge and dermal papilla serve as a niche for hair follicle stem cells, which are characterized by intense proliferative capacity and multipotency. These cells can regenerate not only hair follicles but also sebaceous glands and epidermal cells.^106^ It has been suggested that these stem cells undergo reactivation to replace inactive and atrophic hair follicles.^107^ The dermal papillae, which house these stem cells, are surrounded by blood vessels. Additionally, sebaceous glands are highly vascularized and secrete sebum in an energy-dependent manner, reflecting the organism's metabolic status.^108^ The hair follicle (papilla) contains an inner root sheath, known for its high proliferative capacity^109^ (Fig. 10(I-C)). The outer root sheath is a multilayered tissue predominantly composed of undifferentiated keratinocytes. The inner root sheath terminates in the isthmus region, while the infundibulum contributes to keratin production (Fig. 10(I-D)).^59^

Immunobiological identification of micro vessels was performed using CD31 antibodies, which target platelet/endothelial cell adhesion molecule 1 (PECAM1).^60^ Ki-67 antibodies, which bind to the cell nucleus, were used as markers for cell proliferation during the S-phase and other stages of the cell cycle.^109^ Immunoreaction studies indicated that CD31 antibodies react with micro vessels surrounding the hair sheath (Fig. 10(II-A)) as well as the dermal papillae (Fig. 10(II-B)). Notably, Ki-67 antibodies exhibit a strong reaction with the cells of the inner root sheath at the dermal lamellae. Additionally, staining images of the dermal papilla invagination showed intense reactions at the matrix rim opening, where the inner root sheath originates. The dynamic process of layer formation is illustrated in the transition from the globular papilla to the invaginated dermal papilla structure (Fig. 10(II-C) to Fig. 10(II-D)).^110^

### 3.6. Metabolic energy consumption of human epidermal keratinocytes *in vitro*

Hair shaft formation, hair growth, and hair differentiation are driven by keratinocyte stem cell progeny, or follicular keratinocytes, which originate from the dermal papilla and surround the hair bulb.^111^ Given the complexity of this structure, metabolic energy is essential. This study confirms that polyP serves as an energy source for hair formation.

#### 3.6.1. Effect of Na-polyP-GMP and Na-polyP-COM on keratinocyte growth

**Fig. 11:**
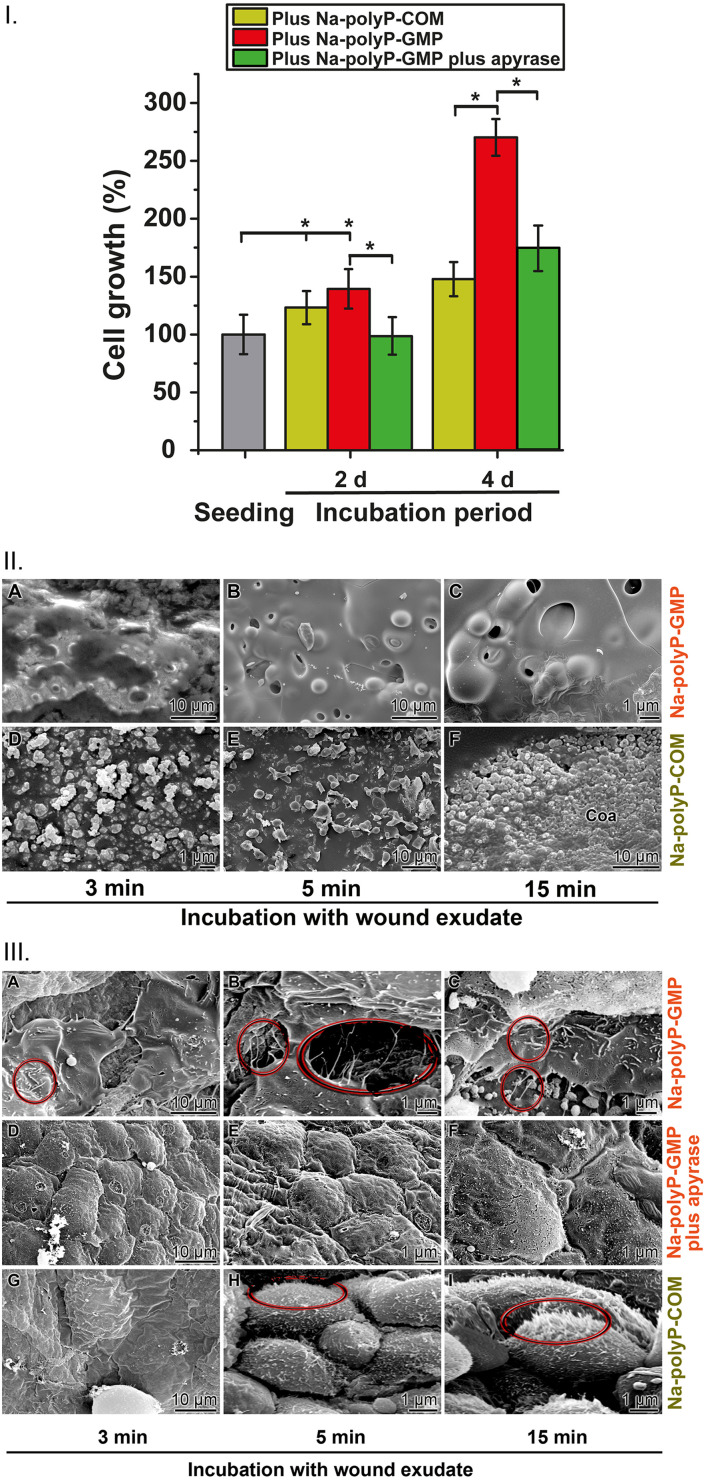
Metabolic energy requirements during growth of keratinocytes in human wound exudate. (I.) The growth of keratinocytes is strongly accelerated by 60 µg mL^−1^ Na-polyP. Keratinocytes were exposed either to Na-polyP-COM or to the Na-polyP-GMP formulation; the experiments were performed using the MTT assay. Besides of obvious acceleration of keratinocyte growth by Na-polyP-GMP, the co-incubation experiment of Na-polyP-GMP with the ATP-depleting enzyme apyrase leads to a significant reduction in the growth potency. This effect is particularly drastic after an incubation period of 4 d; significance *p* < 0.05. (II.) Different potencies of Na-polyP-GMP and Na-polyP-COM in coacervate formation. (A to C) Rapid coacervation of Na-polyP-GMP in the presence of Ca^2+^ and peptides present in the wound exudate. While a smooth surface layer forms here, (D to F) the Na-polyP-COM formulation allows only an initial phase-phase separation, and granules are left in the aqueous droplet phase during the first 15 min exposure. (III.) (A to C) Keratinocytes form elongated microvilli on a Na-polyP-GMP coacervate (circled). After co-incubation with the ATP-eliminating enzyme apyrase (D to F), these protrusions are not formed. (G to I) In contrast, keratinocytes growing on a Na-polyP-COM coacervate form only rudiments of microvilli.

In the presence of Ca^2+^ and peptides, polyP undergoes a phase transition to a coacervate state.^27^ In this phase, the polymer exhibits functional activity.^28^ It generates metabolic energy in the form of ATP through the stepwise enzymatic cleavage of energy-rich P–O–P bonds by the membrane-associated enzymes ALP and subsequently ADK.^36^ The effect of the Na-polyP-GMP formulation on keratinocyte growth was examined in comparison to Na-polyP-COM using the MTT assay (Fig. 11(I)). After two days of incubation, both formulations (60 µg mL^−1^) significantly increased cell growth by 20–40%. However, after a 4 day incubation period, a much stronger effect was observed for the Na-polyP-GMP formulation. Co-addition of the ATP-eliminating enzyme apyrase, which hydrolyzes ATP, resulted in a 35% reduction in cell growth in the Na-polyP-COM experiments (not shown). After four days, the cells exposed to Na-polyP-GMP even exhibited a reduction by 28% in cell growth. This result suggests that ATP regulates cell growth significantly more strongly in the Na-polyP-GMP system compared to the Na-polyP-COM system.

#### 3.6.2. Potency of the two formulations in inducing coacervation

Coacervation typically takes about 60 minutes to complete. To determine if this transition also occurs in wounds, particularly in serous wound fluid, an artificial formulation (from Biochemazone), supplemented with 60 µg mL^−1^ of Na-polyP-GMP or Na-polyP-COM was used. The coacervate phase was examined after 15 minutes using SEM (Fig. 11(II)). The Na-polyP-GMP formulation reached a smooth aqueous droplet phase within 15 minutes (Fig. 11(II-A–C)), whereas Na-polyP-COM remained granular and showed only an initial transition to a gelatinous phase (Fig. 11(II-D–F)).

#### 3.6.3. Morphology of keratinocytes on the coacervate layer

Activated keratinocytes form prominent microvilli upon attachment to a hyaluronan surface.^112,113^ A similar texture has been observed in polyP-driven coacervation phases after 60 minutes of incubation.^28^ On polyP-based surfaces, ATP release during enzyme-coupled polymer degradation *via* ALP and ADK induces keratinocyte microvilli formation.^63^ Electron microscopic ESEM inspections confirmed that cells growing on Na-polyP-GMP developed numerous microvilli (Fig. 11(III-A–C)), whereas those on Na-polyP-COM showed minimal microvilli (Fig. 11(III-G–I)). Further evidence that ATP induces microvilli formation was provided by experiments in which keratinocytes grown on Na-polyP-GMP were co-incubated with apyrase (Fig. 11(III-D–F)).

### 3.7. Successful healing of a bacterially infected wound in a German shepherd

Standard treatments for canine wounds involve antiseptic or antibiotic ointments, but in the case presented, these treatments failed. Furthermore, traditional wound healing methods are known to increase antibiotic resistance, inhibit cell division, or suppress the immune system, and thus possibly lead to chronic wounds.^114–116^

It is very difficult to establish a rational connection between chronic wounds in dogs and humans. Chronic wounds in dogs are usually caused by physical factors such as bites or pressure points from ill-fitting harnesses/bandages, which are only hardly found in human chronic wounds, as well as by burns and wound infections after surgery.^117^ Therefore, it was also difficult to enrol a larger group of dogs in the study. However, one report convinced us also to include a dog in this comparative study. In this study, it was shown that monocytes and macrophages elicit the same healing potential as blood platelets, which are the primary source for polyP in humans.^118^ Platelet-rich fibrin (PRF) is a current research topic due to its regenerative effects in humans.^119^

Standard approaches to heal the chronic wound in the selected dog failed. Therefore, the physician proposed in an open trial the application of Na-polyP-GMP for the healing as a proof-of-concept-study. The rational to apply Na-polyP-GMP also in a dog case came also from the above outlined differences in the wound healing kinetics between human and animal/dog lesions, especially with respect to myofibroblasts function and role during the process of human and dog healing. Especially the differences in the activity and elimination of cellular debris are noteworthy.

In view of this failure in pretreatment and the lack of further treatment methods, a polyP-based energy delivery was considered as a promising alternative. A Na-polyP-GMP hydrogel (300 µg g^−1^) was applied, creating a moist environment that promotes wound healing.^57^ In this environment, Na-polyP-GMP undergoes coacervation, exhibiting self-cleaning properties.^120^

**Fig. 12:**
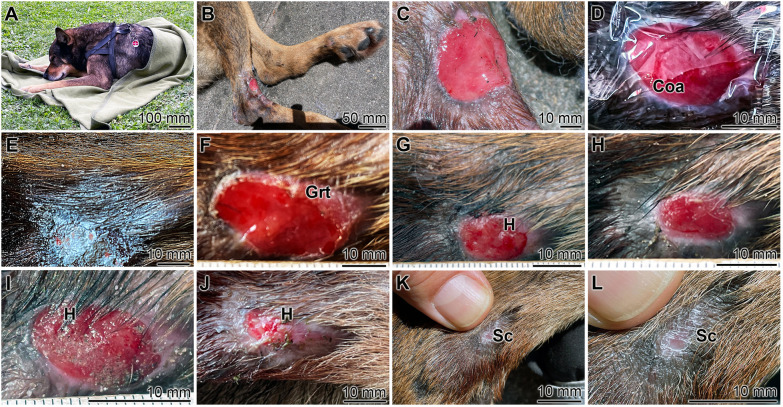
Complete healing process of an infected chronic wound in a dog over a period of 35 days. (A) The animal is a 60 cm tall male German Shepherd. (B) Wound on the right posterior hind limb. (C) Start of the treatment with the Na-polyP-GMP hydrogel. (D) Coacervation (Coa) at the surface of the wound after addition of the Na-polyP-GMP hydrogel. (E) During drying of the hydrogel, granulated polyP particles appear. (F) As treatment progresses, at least every third day with fresh addition of the Na-polyP-GMP hydrogel, the wound size undergoes reduction, and the regeneration zone at the rim forms with granulation tissue (Grt). (G and H) Further reduction of the wound size on day 7 and hair (H) growth at the wound bed. (I) Continued intensive hair growth. (J) Integration of rudimentary stubble into the granulation tissue. (K and L) Formation of wound scab (Sc) followed by normal hair growth and adjusted normal hair density.

The healing of the chronic wound that persisted for 60 days was monitored. Initially, the wound on the dog's right posterior hind limb measured ≈28 mm (Fig. 12A–C). Treatment began on July 21st (Fig. 12D) with a 2 mm-thick hydrogel layer. The gel surface formed a coacervate (Fig. 12D), while the peripheral region dried, leaving behind granular hydrogel. The wound remained in an aqueous phase (Fig. 12E) and was covered with a cotton dressing, which was replaced every three days. By July 27th, the wound had significantly reduced to 14 mm (Fig. 12G and H), with a 2 mm rim of granulation tissue. After another five days, the wound size decreased to 10 mm. Seven days later, new hair began to grow within the wound bed (Fig. 12H–I). By August 25th, after 35 days of Na-polyP-GMP hydrogel application, the wound had fully closed (Fig. 12K and L), allowing the dog to resume swimming therapy. Normal hair growth and density were restored.

## 4. Discussion

Among wounds, chronic wounds in particular are associated with severe morbidity and high costs due to the prolonged treatment duration, making them a significant socio-economic burden.^121^ Despite notable progress in the management of chronic wounds, a definitive breakthrough has yet to be achieved. It has been suggested that activation of the Wnt5a pathway, which is linked to enhanced metabolic energy supply and consumption, can improve and accelerate chronic wound healing.^122^ However, until the introduction of Na-polyP, no suitable energy-generating metabolite had been identified.

**Fig. 13:**
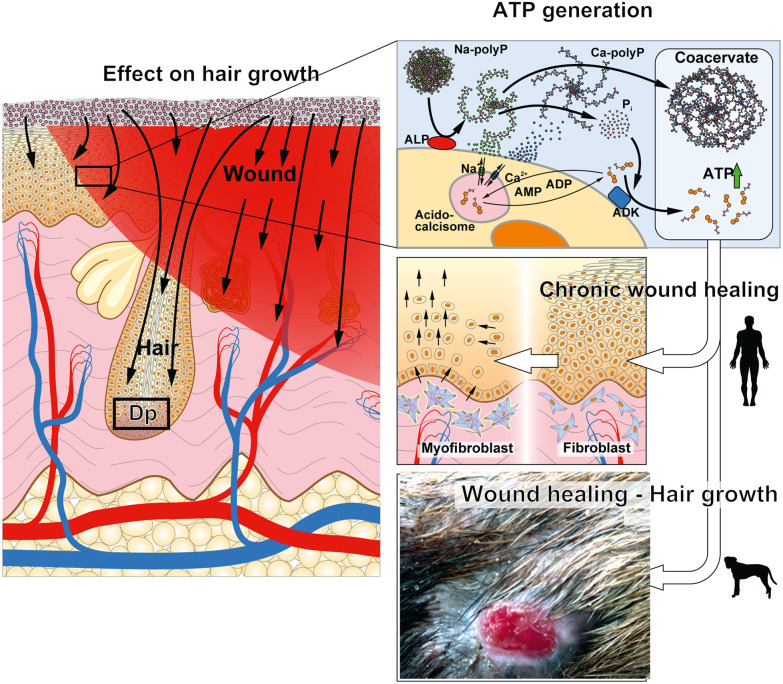
Skin regeneration by Na-polyP-GMP. Left panel: Application of Na-polyP-GMP to speed-up hair growth; the stem cells in the dermal papilla (Dp) show increased proliferation and differentiation. Right panel: The ATP generation process is outlined with the transformation of Na-polyP-GMP into the coacervate phase, allowing the enzymatic release of ATP *via* the membrane-associated enzymes ALP and ADK. PolyP is formed in the acidocalcisome-like dense grana of the blood platelets. The released ATP enables the successful healing of chronic wounds in humans, particularly through the development of fibroblasts into myofibroblasts, as well as the healing of bacterially infected wounds in animals (dog).

Chronic wounds develop due to abnormalities in tissue regeneration following injuries, often caused by insufficient blood supply (Fig. 13). The dominant circulating cells, platelets, contain polyP as a key component. This polymer serves as a condensed energy storage, which is released through sequential enzymatic metabolism and degradation by ALP, followed by phosphorylation of ADP to ATP *via* ADK.^36^ ATP, as a form of metabolic energy, accelerates wound healing, particularly by promoting the differentiation of fibroblast precursors into myofibroblasts^5^ (Fig. 13). Myofibroblasts play a crucial role in producing and organizing the extracellular matrix, which is essential for restoring tissue integrity after injury. These cells also consume significant amounts of ATP^123^ to maintain cellular metabolism, particularly for myofibroblast contraction, which is regulated by the AMPK and mTORC signaling pathways.^36^

PolyP, synthesized from mitochondrial ATP and stored in platelets—presumably within the dense granules—is distributed throughout the bloodstream *via* macrophages.^21^ This suggests that physiologically synthesized polyP is transported to those sites where tissue repair and regeneration are needed. In platelets, two distinct polyP pools exist, depending on the extraction method: a shorter fraction (60–90 P_*i*_ units, obtained *via* phenol/chloroform extraction) and longer polymers (≈400 P_*i*_ units, isolated *via* anion-exchange purification).^124^ Notably, the physiological distribution of polyP chain lengths is relatively narrow.^124,125^ The Na-polyP-GMP formulation presented here successfully achieves this narrow size distribution. Its FTIR spectrum exhibits sharper signal peaks, and XRD analysis confirms its non-crystalline nature—contrary to the Na-polyP-COM formulation, which displays distinct crystalline peaks.

Additionally, the Na-polyP-GMP preparation maintains the pH of the cell culture medium/serum, whereas Na-polyP-COM causes a pH drop, thereby reducing its cell proliferation potency. The impact of the increased ATP environment due to Na-polyP-GMP is evident when co-incubated with apyrase, an ATP-degrading enzyme. In the presence of apyrase, keratinocyte proliferation is drastically impaired, and their microvilli formation—essential for absorption, adhesion, and communication—is severely hindered. In contrast, Na-polyP-COM only supports rudimentary and small microvilli formation.

Since 1992, it has been reported that activated platelet supernatant significantly accelerates wound healing, a phenomenon attributed to its high concentration of growth factors such as PDGF.^126^ Our group later discovered that polyP itself is a key factor behind this effect, as it serves as a precursor for ATP production in the wound bed *via* the enzymes ALP and ADK on the surface of keratinocytes.^63^

Both chemical-analytical and *in vitro* cell culture data indicate the superiority of Na-polyP-GMP over Na-polyP-COM in promoting tissue regeneration. The new formulation exhibits significantly higher proliferative potential in both rapidly differentiating SaOS-2 cells and keratinocytes. Enhanced differentiation and proliferation—crucial during the transition from the inflammatory to proliferative phases of wound healing and during myofibroblast differentiation—make Na-polyP-GMP particularly effective.^127^

For the *in vivo* studies in this paper, continuing our previous studies on patients with this polymer, we chose two chronic wounds humans^57,63^ and a persistently infected wound in a dog (Fig. 13). One reason for this is that wound healing dynamics differ between these species: dogs generally heal faster due to a more robust immune response and thicker, more elastic skin with higher collagen content, though this also increases the susceptibility to infections.^117^ In dogs, granulation tissue forms rapidly, whereas in humans, it develops more slowly and often necessitates skin grafts.^128^ These human and animal are considered as feasibility studies under the control of physicians to prove the efficiency of the new polymer.

In these studies, the sodium salt of polyP (Na-polyP) was taken due to its high water solubility, mild hydration properties, and suitability for use as a polyP hydrogel, which benefits from phase transition during coacervation.^28,63^ The human case study involved a patient with a chronic venous ulcer caused by poor leg vein circulation—a condition frequently associated with diabetes^129^—and a patient with a small but therapy-resistant traumatic ulcer. Hydrocolloid dressings can aid acute wound healing and provide some benefit for chronic wounds.^130^ Progress has been made in the treatment of chronic wounds through the use of autologous platelet-rich plasma.^131^ Since polyP has been identified as a major regenerative component of platelets, direct application of the polymer has been proposed. Using pure polyP allows for precise quantification, standardization, and formulation as a hydrogel or collagen matrix.^57,63^ Additionally, Na-polyP-GMP is GMP-compliant, infection-free, and cost-effective. Treatment with the Na-polyP hydrogel enabled complete healing of the chronic venous ulcer and the therapy-resistant traumatic ulcer presented here as examples of a chronic wound within two to five months.

Wound healing and hair growth share common biological mechanisms.^132^ Recent studies indicate that hair follicle stem cells share properties with wound-healing stem cells due to their multipotent/pluripotent nature and high proliferation rates, driven by the Wnt/β-catenin signaling pathway.^133^ Fibroblasts, specialized mesenchymal stem cells, contribute to collagen deposition in both processes.^134^ Both regeneration processes are highly energy-dependent (Fig. 13) and impaired in diabetic patients, who exhibit reduced skin self-renewal and hair follicle activation.^135^ In diabetic mice, Na-polyP-GMP significantly promoted hair papillae formation near wounds, enhancing hair density. The *ex vivo* studies with human explants showed that Na-polyP-GMP treatment increased vascularization and stem cell proliferation, as indicated by CD31 and Ki-67 immunostaining.

The results of these case studies confirm the high regenerative potential of Na-polyP-GMP in clinical applications, supporting its role in both wound healing and hair growth.

Finally, it is imperative to highlight that polyP has been clinically proven to be safe^57,63,136^ and classified as harmless by both the U. S. Food and Drug Administration (FDA) and the European Union (EU).^137^ This physiological polymer has been assigned the E-numbers E452i for Na-polyP, E452iii for Na/Ca-polyP, and E452iv for Ca-polyP.^137^ In addition, polyP showed no carcinogenicity, reproductive or developmental toxicity, and no significant genotoxicity in the Ames test using *Salmonella typhimurium*.^138^ The polymer also has a very high LD_50_ value, exceeding 2000 mg kg^−1^.^139^ Furthermore, a standardized test evaluating polyP for skin irritation and sensitization was conducted according to OECD guidelines (OECD 2002 – Test No. 404) using New Zealand white rabbits.^140^ No adverse reactions were observed in the animals after direct application of polyP to the test sites.

The exceptional property of polyP, and a major reason for the strong regenerative and healing properties of this polymer, lies in its ability to store and release metabolic energy. This occurs through the sequential enzymatic cleavage of its phosphoanhydride bonds by ALP, followed by the phosphorylation of the resulting ADP to generate ATP—an essential energy carrier and signaling molecule.^36,141^ ATP serves as a crucial metabolic energy source, supporting anabolic reactions through the triad of “metabolic energy – mechanical energy – heat”.^5^ Myofibroblasts, which are primarily located at the periphery of wounds and crucially involved in tissue remodeling during granulation, are likely the strongest drivers of polyP-dependent wound regeneration. The polymer's regenerative effects are further enhanced by its ability to interact with platelets and modulate the immune system, including the innate immune response.^83,142^ For example, bacterial polyP with a chain length of 700 P_*i*_ units has been reported to inhibit interferon production and its associated signaling pathways.^143^ It should be noted, however, that the biological effects of polyP depend on the polymer chain length. The same publication showed that, in contrast to long-chain polyP, shorter chains with 14 and 100 P_*i*_ units do not have a significant inhibitory effect on the activity of the interferon regulatory pathway.^143^

The availability of GMP-compliant polyP, as described in our study, is also of interest for various other clinical applications, for example as a component of bone cements in surgery or dentistry,^144^ or in antiviral therapy.^145^

## 5. Conclusions

The results of this study suggest that the growth- and differentiation-promoting effects of polyP can be further enhanced by using polyP preparation with a narrow size distribution of the polymer chains of around 30 P_*i*_ residues, which does not alter the pH of the incubation medium. The newly developed polyP formulation was not only highly effective in accelerating the healing of chronic wounds in humans but, for the first time, also indicated efficacy in a domestic animal—a German Shepherd dog—with a slow-healing, bacterially infected wound. Furthermore, the polyP preparation exhibited a strong stimulatory effect on hair growth, further expanding the potential applications of the polymer. The chain length of the Na-polyP-GMP matches the P_*i*_ length of the physiological polymer and is therefore highly tissue- and biocompatible.

## Author contributions

WEGM: conceptualization, data curation, formal analysis, funding acquisition, investigation, methodology, project administration, resources, supervision, writing – original draft, writing – review & editing; MN: conceptualization, data curation, formal analysis, methodology, writing – original draft, writing – review & editing; XQL: data curation, formal analysis, methodology, writing – original draft, writing – review & editing; HN: conceptualization, data curation, formal analysis, investigation, methodology, visualization, writing – original draft, writing – review & editing; MB: data curation, formal analysis, investigation, methodology, validation, visualization, writing – original draft, writing – review & editing; RD: conceptualization, formal analysis, methodology, resources, writing – original draft, writing – review & editing; RM: data curation, formal analysis, methodology, writing – original draft, writing – review & editing; CXW: conceptualization, formal analysis, methodology, writing – original draft, writing – review & editing; HU: conceptualization, formal analysis, methodology, writing – original draft, writing – review & editing; HCS: conceptualization, formal analysis, funding acquisition, methodology, project administration, supervision, writing – original draft, writing – review & editing; XHW: conceptualization, data curation, formal analysis, funding acquisition, investigation, methodology, project administration, resources, supervision, writing – original draft, writing – review & editing.

## Conflicts of interest

There are no conflicts to declare.

## Supplementary Material

BM-014-D6BM00151C-s001

## Data Availability

Supplementary information (SI) is available. See DOI: https://doi.org/10.1039/d6bm00151c.
