# Supplementary material for: A GMP-compliant formulation of regeneratively active polyphosphate for wound healing and skin regeneration
Source: Biomater Sci. 2026 Apr 24;14(12):3181–99. doi: 10.1039/d6bm00151c (PMC13139896; doi:10.1039/d6bm00151c)
Supplement: BM-014-D6BM00151C-s001 [file BM-014-D6BM00151C-s001.pdf]

Supplementary Information (SI) for Biomaterials Science.
This journal is © The Royal Society of Chemistry 2026

Supplementary information to the article:

A GMP-compliant formulation of regeneratively active polyphosphate for wound healing and skin regeneration

Werner E.G. Müller*[a], Meik Neufurth[a], Xiaoqin La[b], Hadrian Nassabi[c,d], Mathias Brösicke[e], Rita Dobmeyer[f], Rafael Muñoz-Espí[g], Changxin Wu[b], Hiroshi Ushijima[h], Heinz C. Schröder[a] and Xiaohong Wang*[a]

[a] ERC Advanced Investigator Grant Research Group at the Institute for Physiological Chemistry, University Medical Center of the Johannes Gutenberg University, Duesbergweg 6, D-55128 Mainz, Germany E-mail: wmueller@uni-mainz.de (W.E.G. Müller); wang013@uni-mainz.de (X.H. Wang)
[b] Institutes of Biomedical Sciences, Key Laboratory of Chemical Biology and Molecular Engineering of Ministry of Education of China and Key Laboratory of Medical Molecular Cell Biology of Shanxi Province, Shanxi University, No. 92 Wucheng Road, 030006 Taiyuan, China
[c] Department of Dermatology with Plastic Surgery, SRH Wald-Klinikum Gera GmbH, Friedens Street 122, D-07548 Gera, Germany
[d] Department of Dermatology, University Medical Center of the Johannes Gutenberg University, Langenbeck Street 1, D-55131 Mainz, Germany
[e] Academy of Non-Profit Sciences in Erfurt, Gotthardt Street 21, 99084 Erfurt, Germany
[f] Galenus GH AG, Rain Street 7, 6052 Hergiswil, Switzerland
[g] Institute of Materials Science (ICMUV), Universitat de València, C/Catedràtic José Beltrán 2, 46980 Paterna - València, Spain
[h] Division of Microbiology, Department of Pathology and Microbiology, Nihon University-School of Medicine, 30-1 Oyaguchi-Kamicho, Itabashi-Ku, 173-2610, Tokyo, Japan

*** Corresponding authors:**
Prof. Dr. X.H. Wang and Prof. Dr. W.E.G. Müller,
ERC Advanced Investigator Grant Research Group at Institute for Physiological Chemistry, University Medical Center of the Johannes Gutenberg University, Duesbergweg 6, D-55128 Mainz, Germany. Tel.: +49 6131-39-25910; Fax: +49 6131-39-25243; E-mail: wmueller@uni-mainz.de (Prof. Dr. W.E.G. Müller); wang013@uni-mainz.de (Prof. Dr. X.H. Wang).

Biomarker to measure the superior efficiency of Na-polyP-GMP versus Na-polyP-COM

Only recently, we had Na-polyP-GMP ready for use in wound healing. To assess the differences in healing potency between Na-polyP-COM and the advanced Na-polyP-GMP formulation, we investigated the potency of the effect of both polyP preparations on myofibroblasts. The characteristic feature of myofibroblasts is their contraction property, which is dependent on ATP supply.[1] During the differentiation of fibroblasts to myofibroblasts, the cells elongate and develop a well-developed contractile apparatus based on robust actin stress fibers.[1,2] These stress fibers respond to ATP and have a diameter of 1.0 µm, each and reach lengths of up to 100 µm. Due to the known property of both Na-polyP-GMP and Na-polyP-COM to serve as a source for ATP generation, we measured the length of all the stress fibers in stained sections with myofibroblasts. Of course, the 9 patients treated so far are not enough for regulatory approval of polyP as a medical device.[3-5] However, the formulation of the GMP-conform active ingredient Na-polyP-GMP with the beneficial properties summarized above was a lengthy and arduous process and can be considered as an important step towards an API (Active Pharmaceutical Ingredient) acceptance. The GMP certificate for Na-polyP-GMP was granted by the EMA authority.[2]

The superior efficiency of the Na-polyP-GMP formulation can also be deduced from the present clinical results of the proof-of-concept open studies. It is evident that the mean healing time for all patients studied to date is 6.33 ± 2.06 weeks – a remarkably short period.

Extracellular ATP is a "danger signal" that binds to P2 purinergic receptors (especially on P2Y2 and P2X7) on myofibroblasts.[6,7] This triggers a signaling cascade that involves an increase in intracellular calcium and activation of the RhoA/ROCK pathway, which is the master regulator of actin polymerization and myosin contractility. Extracellular ATP promotes the assembly and stabilization of pre-existing stress fiber components via the RhoA signaling pathway.[8] Already previously, we had proposed that the transition of fibroblasts to myofibroblasts is triggered by ATP, which is produced after enzymatic digestion of polyP with ALP and ADK.[8,9] Therefore, we used the length of the myofibroblasts obtained from sections through the granulation tissue. The length of the stress fibers is correlated with the length and differentiation status of the myofibroblasts.[9-12] , caused by the exogeneous ATP.

For this study, cells obtained from biopsies of chronic wounds of patients, described here not treated with Na-polyP-GMP were compared with those obtained from wounds freshly treated Na-polyP-GMP (after an application period of 12 h). To highlight the different cell morphologies, the same antibody was used for the reaction with human α smooth muscle actin.

However, in the polyP-treated specimens, the immunocomplexes were visualized in red with a goat anti-Alexa Fluor-350 secondary antibody (Fig. S-2 (I-A)), while the immunocomplexes in the polyP-untreated slices were visualized in green with a FITC-labeled secondary antibody (Fig. S-2 (I-B)). In the latter sections, it is evident that the cytoplasm of the fibroblasts also contains a cytoskeleton, which is colored differently due to the actin fibers present in these precursor cells.[9]

For quantification, tissue sections were prepared and analyzed from patients treated with Na-polyP-COM and Na-polyP-GMP. The length of the fibroblasts in the Na-polyP-GMP-treated wound examined here was compared with the length of the cells in the Na-polyP-COM-treated wounds (Fig. S-1). The result showed that the mean size of the myofibroblasts in the Na-polyP-GMP-treated chronic wound was significantly larger than the size of the myofibroblasts in the Na-polyP-COM-treated wounds (Fig. S-2 (II)).

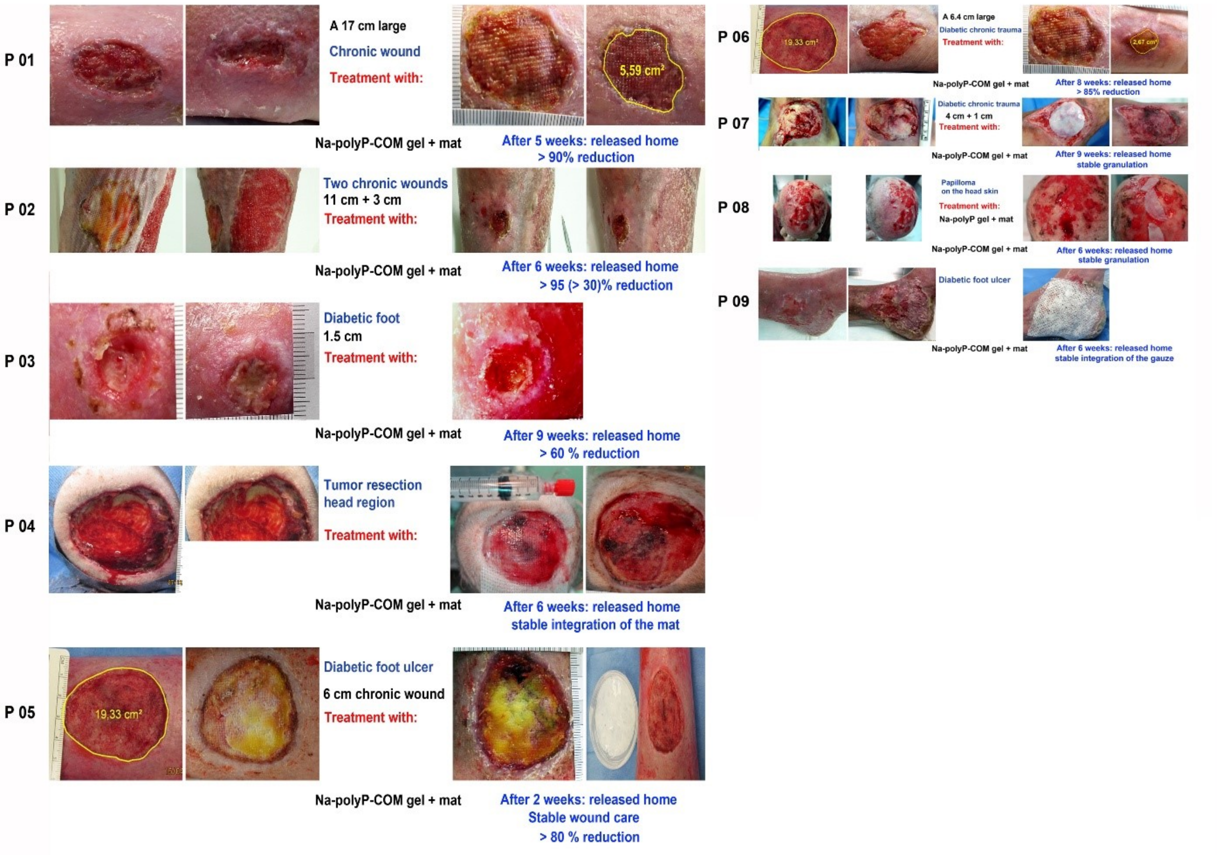

Fig. S-1. Complete series of Na-polyP-COM-treated patients in the proof-of-concept study. Previously described cases (images with permissions). All 9 patients (Patient P 01 to P 09) had been treated with Na-polyP-COM.

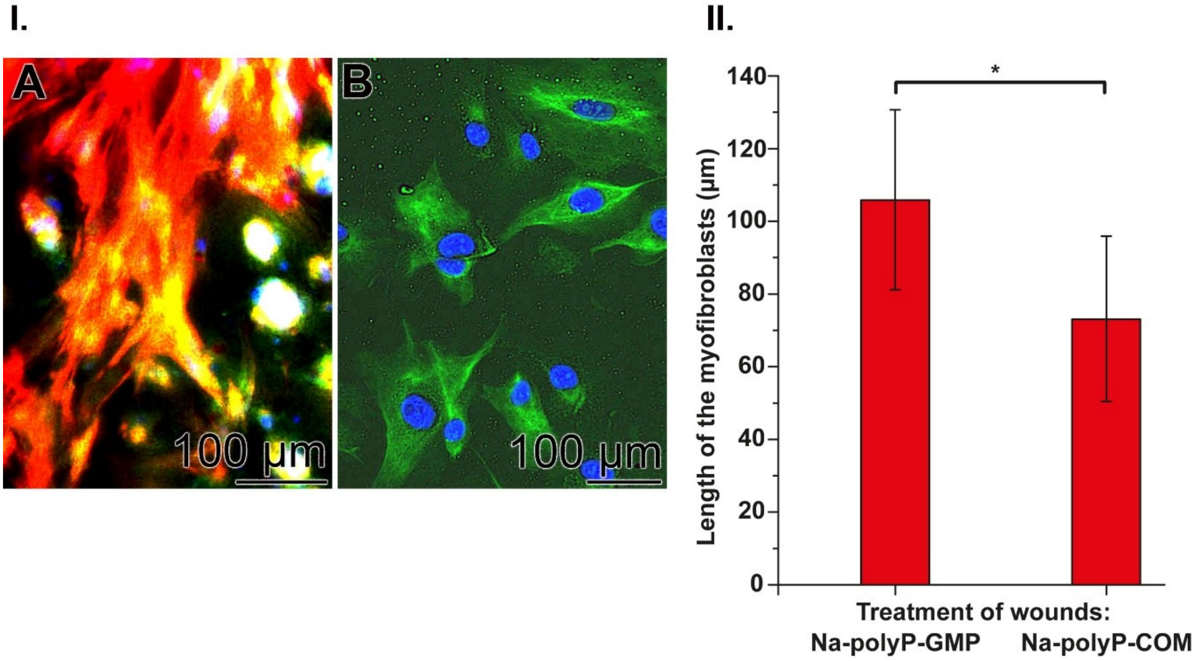

Fig. S-2. (**I.**) (**A**) During the exposure of the wounds to Na-polyP-GMP, the fibroblasts progress to the myofibroblast stage. Anti-human-α smooth muscle actin antibodies were used to identify the stress fibers in these cells. In (A), the immunocomplexes in the Na-polyP-GMP-treated wounds were visualized with anti-Alexa Fluor-350 secondary antibodies (in red). In wounds not treated with Na-polyP-GMP, the immunocomplexes were counterstained with a FITC-labeled secondary antibody (in green). In the latter case, the cytoplasmic actin structures in the fibroblasts (not the stress fibers) are also visualized by more intense coloration. (**II.**) Change in the length of the cells in the Na-polyP-GMP-treated wound; the chronic wound case documented here was chosen. The size of the fibroblasts in the Na-polyP-COM-treated wounds from the patients shown in Figure S-1 was determined. The correlation coefficient is significant ($p < 0.05$).

References

1 S. A. Alkaabi, D. S. N. Kalla, G. A. Alsabri, A. Fauzi, N. Jansen, A. Tajrin, M. Neufurth, H. C. Schröder, X. H. Wang and W. E. G. Müller, *Pilot and Feasibility Studies*, 2021, **7**, 199.

2 European Parliament and Council of the European Union, *Official Journal of the European Union*, 2008, **L354**, 16.

3 W. E. G. Müller, H. Schepler, M. Neufurth, R. Dobmeyer, R. Batel, H. C. Schröder, X. H. Wang, *Theranostics*, 2024, **14**, 5262–5280.

4 W. E. G. Müller, H. Schepler, M. Neufurth, S. Wang, V. Ferrucci, M. Zollo, X. H. Wang, *Journal of Materials Science & Technology*, 2023, **135**, 170–185.

5 H. Schepler, M. Neufurth, S. Wang, Z. She, H. C. Schröder, X. H. Wang and W. E. G. Müller, *Theranostics*, 2022, **12**, 18–34.

6 A. Noom, B. Sawitzki, P. Knaus and G. N. Duda, *NPJ Regenerative Medicine*, 2024, **9**, 15.

7 N. Sandbo and N. Dulin, *Translational Research*, 2011, **158**, 181–196.

8 G. Isenberg, P. C. Rathke, N. Hülsmann, W. W. Franke and K. E. Wohlfarth-Bottermann, *Cell and Tissue Research*, 1976, **166**, 427–443.

9 N. Riteau, P. Gasse, L. Fauconnier, A. Gombault, M. Couegnat, L. Fick, J. Kanellopoulos, V. F. Quesniaux, S. Marchand-Adam, B. Crestani, B. Ryffel and I. Couillin, *American Journal of Respiratory and Critical Care Medicine*, 2010, **182**, 774–783.

10 B. C. Willis, R. M. duBois and Z. Borok, *Proceedings of the American Thoracic Society*, 2006, **3**, 377–382.

11 L. Rosso, B. Peteri-Brunbäck, V. Vouret-Craviari, C. Deroanne, J. D. Troadec, S. Thirion, E. Van Obberghen-Schilling and J. M. Mienville, *Glia*, 2002, **38**, 351–362.

12 Y. Tai, E. L. Woods, J. Dally, D. Kong, R. Steadman, R. Moseley and A. C. Midgley, *Biomolecules*, 2021, **11**, 1095.
